# Drug repurposing for cancer treatment through global propagation with a greedy algorithm in a multilayer network

**DOI:** 10.20892/j.issn.2095-3941.2020.0218

**Published:** 2022-01-15

**Authors:** Xi Cheng, Wensi Zhao, Mengdi Zhu, Bo Wang, Xuege Wang, Xiaoyun Yang, Yuqi Huang, Minjia Tan, Jing Li

**Affiliations:** 1 Department of Bioinformatics and Biostatistics, School of Life Sciences and Biotechnology, Shanghai Jiao Tong University, Shanghai 200240, China; 2 The Chemical Proteomics Center and State Key Laboratory of Drug Research, Shanghai Institute of Materia Medica, Chinese Academy of Sciences, Shanghai 201203, China; 3 University of Chinese Academy of Sciences, Beijing 100049, China; 4 Key Laboratory of Tissue Microenvironment and Tumor, Shanghai Institute of Nutrition and Health, Shanghai Institutes for Biological Sciences, University of Chinese Academy of Sciences, Chinese Academy of Sciences (CAS), Shanghai 200031, China

**Keywords:** Database, drug repurposing, machine learning, network random walk, proteomics analysis

## Abstract

**Objective:**

Drug repurposing, the application of existing therapeutics to new indications, holds promise in achieving rapid clinical effects at a much lower cost than that of *de novo* drug development. The aim of our study was to perform a more comprehensive drug repurposing prediction of diseases, particularly cancers.

**Methods:**

Here, by targeting 4,096 human diseases, including 384 cancers, we propose a greedy computational model based on a heterogeneous multilayer network for the repurposing of 1,419 existing drugs in DrugBank. We performed additional experimental validation for the dominant repurposed drugs in cancer.

**Results:**

The overall performance of the model was well supported by cross-validation and literature mining. Focusing on the top-ranked repurposed drugs in cancers, we verified the anticancer effects of 5 repurposed drugs widely used clinically in drug sensitivity experiments. Because of the distinctive antitumor effects of nifedipine (an antihypertensive agent) and nortriptyline (an antidepressant drug) in prostate cancer, we further explored their underlying mechanisms by using quantitative proteomics. Our analysis revealed that both nifedipine and nortriptyline affected the cancer-related pathways of DNA replication, the cell cycle, and RNA transport. Moreover, *in vivo* experiments demonstrated that nifedipine and nortriptyline significantly inhibited the growth of prostate tumors in a xenograft model.

**Conclusions:**

Our predicted results, which have been released in a public database named The Predictive Database for Drug Repurposing (PAD), provide an informative resource for discovering and ranking drugs that may potentially be repurposed for cancer treatment and determining new therapeutic effects of existing drugs.

## Introduction

Drug repurposing applies existing therapeutics to new clinical indications^1^. This time-saving, cost-efficient, and low-risk approach is increasingly being used for drug discovery and development^2^. Prior drug-repurposing successes span multiple disease areas. For example, sildenafil, a classical phosphodiesterase inhibitor, was the first Food and Drug Administration (FDA)-approved oral therapy for erectile dysfunction^3^. Canakinumab, which was originally used to treat cryopyrin-associated periodic syndromes, is expected to be commercialized in the market for the treatment of cardiovascular diseases, owing to its beneficial effects on recurrent cardiovascular events^4^. Warfarin, a drug used to prevent blood clots, has recently been shown to decrease cancer incidence in older patients^5^, thus suggesting that it may be an inexpensive means of inhibiting cancer progression. Therefore, many commercially marketed drugs may have unexpected, attractive, and practical effects.

Several methods have been proposed for inferring new indications that may be treated with known drugs, including 3D structural docking simulation, machine learning prediction, and ranking in biological networks^6,7^. However, some methods are limited by the available evidence. By focusing on a specific disease or hundreds of diseases, previous studies have attempted to make predictions by incorporating different types of information into networks, such as drug-target interactions, disease similarities, disease-gene relationships, protein interactions, and TF-target pairs^8–15^.

In this study, by targeting 4,096 diseases, including various cancers listed in the Medical Subject Headings (MeSH) database, and 1,419 drugs in DrugBank, we performed a global drug repurposing prediction analysis by using a greedy algorithm and uncovered new potential therapeutic effects for existing drugs through a global random walk in a multiple-layer heterogeneous network. The links in our network consisted of drug-target interactions, protein-protein interactions, disease-gene relationships, drug-drug similarities, disease-disease similarities, and drug-disease relationships. After evaluation of the overall performance of our network-based prediction model, we selected the top-ranked drug-cancer pairs, validated the effects, and explored the underlying mechanisms by using drug sensitivity experiments and tandem mass tag (TMT)-based quantitative proteomics. Through further *in vivo* experiments, we demonstrated the antitumor effects of novel repurposed drugs in a xenograft model. Finally, we built a public database named The Predictive Database for Drug Repurposing (PAD), which provides a user-friendly interface for ranking queries according to diseases, drugs, or drug-disease pairs. PAD is freely available at http://lilab.life.sjtu.edu.cn:8080/pad/index.html.

## Materials and methods

### Network datasets

Here, we provide a brief description of the datasets and corresponding matrix representation used in this study.

#### Disease-gene interactions

The human disease-gene interactions were extracted from The Comparative Toxicogenomics Database (CTD)^16^. CTD is widely used to illustrate the effects of environmental chemicals on human health at the genetic level. It provides complete and updated data on the associations between genes and diseases. Disease identifiers were obtained from the MeSH database, and gene names were converted into UniProt IDs. Finally, 599,256 curated human disease-gene relationships were obtained.

#### Drug-target interactions

The drug-target interactions were derived from the DrugBank database^17^. The extensive drug and drug-target data in DrugBank have enabled the discovery and repurposing of existing drugs for novel targets or indications^8,9,11^. Here, we focused on only FDA-approved drugs, because their safety is supported. Drugs were included in the analysis only if simplified molecular-input line-entry system (SMILES) structure descriptions were available in DrugBank. Ultimately, 6,994 interactions between 1,606 drugs and 1,966 targets were included.

#### Protein-protein interactions

The human protein-protein interaction data were downloaded from the STRING database^18^. STRING contains updated data on human protein-protein interactions obtained from multiple sources, including experimental data, text mining, and computational prediction. After converting the protein identifiers to UniProt IDs, we obtained 5,319,165 unique interactions among 17,878 proteins.

#### Drug-drug similarity relationships

The drug chemical structure similarity relationships were determined on the basis of the SMILES structural descriptions in DrugBank. Open Babel 2.4.1 was used to determine the similarity between 2 chemical structures. In this software, the molecular fingerprint, referred to as *FP2*, encodes the chemical structure of a molecule. Every molecule with a string length of 1 to 7 can be converted into a binary-encoded string with a length of 1,024 for identification of all linear and ring substructures. Then, the structural similarity of 2 molecules can be measured by using the Tanimoto coefficient, which is defined as the number of common bits divided by the union of the bit set^19^.

#### Disease-disease similarity relationships

The disease-disease similarity relationships were determined according to the classification systems in the MeSH database. The similarity between each pair of diseases was computed with Lin’s node-based similarity method, on the basis of the MeSH identifiers of the diseases. The R package MeSHSim^20^ was used for this process.

#### Drug-disease relationships

The drug-disease relationships were extracted from the KEGG DRUG and KEGG DISEASE databases, which provide data on drug-disease relationships that have been validated at the molecular level. A relationship was included only when both the drug and the disease were identified in our model. A total of 997 relationships between 377 drugs and 308 diseases were retained as the validation set, on the basis of prior knowledge.

### Construction of adjacent matrices

To both focus on key proteins and reduce false results, we rearranged the network of diseases, proteins, and drugs. Tests revealed that against the background of a massive number of interactions, the probability of a walker arriving at a certain node through 3 or more nodes is quite low. Consequently, removing redundant nodes and edges would dramatically decrease the complexity of the model without affecting the prediction. On the basis of this assumption, we retained the proteins on paths with fewer than 3 proteins that linked a disease node and a drug node. Redundant disease and drug nodes without any retained protein neighbors were removed.

Six adjacent matrices were built according to the rearranged information. S_1_, S_2_, S_3_, S_4_, S_5_, and S_6_ represent the disease-gene relationship matrix, drug-target interaction adjacency matrix, protein-protein interaction matrix, drug chemical structure similarity matrix, disease-disease similarity matrix, and drug-disease relation matrix, respectively. The entity S_1_(*i*, *j*) in row *i* of column *j* of S_1_ equals 1 when disease *i* and gene *j* have a verified relationship and otherwise equals 0; S_2_(*i*, *j*) equals 1 if protein *i* is the target of drug *j* and otherwise equals 0; S_3_(*i*, *j*) is the STRING score of the protein *i*-protein *j* interaction; S_4_(*i*, *j*) is the structural similarity ratio of drug *i* and drug *j*, which ranges from 0 to 1; S_5_(*i*, *j*) is the similarity ratio of disease *i* and disease *j*, according to the classification of diseases in the MeSH database; and S_6_(*i*, *j*) equals 1 if drug *i* and disease *j* have a verified relationship, and otherwise equals 0.

### Random walk in the heterogeneous network

The algorithm has been explained in detail previously^8^. Here, we made some changes in the initial random walk process for topological differences.

Previous research^8^ has demonstrated that to predict the potential efficacy of a given drug, the drug itself and its target proteins should all be denoted the seed nodes. Hence, the initial probability of the heterogeneous network can be represented as:

$$p_{0}=\left[\begin{array}{l} au_{0}\\ bv_{0}\\ ch_{0} \end{array}\right]$$

The parameters *a*,*b*,*c* ϵ [0,1] weight the importance of the disease, protein, and drug network, respectively. The sum of *a*, *b*, and *c* is 1. The details of the initial probability setting are illustrated in **Supplementary materials and methods**.

Then, the transition matrix should be selected to implement the random walk. Nine small-scale transition matrices were built to determine the transition probability from one type of node to another. We defined the transition matrix of the heterogeneous network as:

$$M=\left[\begin{array}{l} M_{disease-disease}\;M_{disease-protein}\;M_{disease-drug}\\ M_{protein-disease}\;M_{protein-protein}\;M_{protein-drug}\\ M_{drug-disease}\;M_{drug-protein}\;M_{drug-drug} \end{array}\right]$$

In the formula, *M*_*a–b*_ denotes the probability of the transition from networks a to b. The details of the transition matrix are illustrated in the **Supplementary materials and methods**. In the first strategy, the elements in *M*_*disease–drug*_ and *M*_*drug–disease*_ were set to 0 to avoid the bias of prior knowledge, because our final prediction results are drug-disease relations. However, in the second strategy, the known drug-disease relationships from KEGG were included in the model. Thus, 2 matrices represented the transition matrix from the disease network to the drug network and the matrix from the drug network to the disease network. The transition matrices were defined by the adjacent matrices, as described in the **Supplementary materials and methods**.

A random walk was implemented on the heterogeneous network after the transition matrix *M* was represented as the minimum-maximum normalized matrix to increase the convergence rate in the iterative process. *p_t_* can be denoted as a vector in which the *i*-th element represents the probability of finding the walker at node *i* after step *t*. The parameter *r* is the probability of the walker restarting from the seed nodes. This probability can be calculated iteratively:

$$p_{t+1}=(1-r)M^{T}p_{t}+rp_{0}$$

After sufficient steps, a stable probability *p*_∞_ can be obtained. Here, *p*_∞_ can be represented as:

$$p_{\infty }=\left[\begin{array}{l} au_{\infty }\\ bv_{\infty }\\ ch_{\infty } \end{array}\right]$$

In this study, we considered the probability to converge when the change between *p*_*t*_ and *p*_*t*+1_, as measured by the L_1_ norm, was less than 10^−10^. We expected that the diseases with higher probability in *u*_∞_ would be more likely to be successfully treated by drug *i*. All the datasets and random walk codes can be found in GitHub (https://github.com/Li-Lab-Proteomics/PAD).

### Evaluation of model performance

For each drug-disease relationship in the validation set, we determined the disease prioritization of the drug and reordered the corresponding disease within a list of 99 randomly selected diseases to generate a new ranking ranging from 1 to 100. According to the new rankings, we calculated sensitivity and specificity values. Here, sensitivity refers to the frequency (% of all prioritizations) of the occurrence of the disease in relationships ranked above a particular threshold, whereas specificity refers to the percentage of diseases ranked below the disease within the relationship. To allow for comparison across models with different parameters, we plotted ranking receiver operating characteristic (ROC) curves and the area under curve (AUC) to measure the performance of the model. An AUC value of 100% indicated that every disease within the relationships ranked first, whereas a value of 50% indicated that the disease was ranked randomly. We repeated this process 50 times to obtain the average AUC value to reduce random errors. For comparison, we constructed a random set by replacing the diseases in the validation set with randomly selected diseases and repeating the process.

### Cell culture and reagents

MCF-7 and MDA231-LM2-4175 cells were grown in DMEM supplemented with 4 mM L-glutamine, 4,500 mg/L glucose, 100 U/mL penicillin, 0.1 mg/mL streptomycin, 10% FBS, and sodium pyruvate, whereas LNCaP, DU145, and MGC-803 cells were grown in RPMI-1640 medium supplemented with 2.05 mM L-glutamine, 0.1 mg/mL streptomycin, 100 U/mL penicillin, 10% FBS, and 25 mM HEPES. Dextromethorphan HBr monohydrate (Selleck Chemicals, Houston, TX, USA), tetracycline hydrochloride (J&K Scientific, Beijing, China), nifedipine (Selleck Chemicals, Houston, TX, USA), nortriptyline hydrochloride (Sigma-Aldrich, St. Louis, MO, USA), atorvastatin calcium (Chinese National Compound Library, Shanghai, China), metformin hydrochloride (Sigma-Aldrich, St. Louis, MO, USA), chlorpropamide (Selleck Chemicals, Houston, TX, USA), tolazoline HCl (Selleck Chemicals, Houston, TX, USA), tiaprofenic acid (Selleck Chemicals, Houston, TX, USA), and decamethonium bromide (Selleck Chemicals, Houston, TX, USA) were dissolved in DMSO, and each stock solution was stored at −20 °C. The compound 5-fluorouracil (5-FU) was dissolved in saline solution. C18 Zip Tips were from Millipore Corporation (Billerica, MA, USA). Acetonitrile and formic acid were from Sigma-Aldrich. DMEM and RPMI-1640 medium were from Hyclone (South Logan, UT, USA). Trypsin was from Hualishi Scientific (Beijing, China).

### Cell viability assays

For cell viability assays, 2–6 × 10^3^ cells were seeded in 96-well plates with 100 µL of the relevant medium for 1 day and then subjected to treatment with different drugs for 72 h. Cell counting kit-8 (CCK-8) assays (Dojindo Molecular Technologies Inc., Kumamoto, Japan) were used to measure cell viability, as described previously^21^.

### Preparation of protein whole cell lysates and in-solution tryptic digestion

Protein extraction and in-solution digestion were performed as described as previously^21–23^. Before lysis, cells were washed 3 times with ice-cold Dulbecco’s PBS. The lysis buffer, which consisted of 8.0 M urea in 100 mM NH_4_HCO_3_, pH 8.0, supplemented with 2× protease inhibitor cocktail (Calbiochem, Darmstadt, Germany), was added to resuspend the pellet. After incubation on ice for half an hour, the lysate was sonicated for 4 min to enable complete lysis (2 s of sonication time at 5 s intervals). After centrifugation at 21,130 × g at 4 °C for 15 min, the supernatant was transferred into a new tube. Quantitative analysis was performed with a BCA protein assay kit (Beyotime Biotechnology, Shanghai, China). Before digestion, reduction and alkylation reactions were performed. The protein solution was reduced with 5 mM dithiothreitol and incubated at 56 °C for half an hour, then incubated with 15 mM iodoacetamide at 25 °C in the dark for half an hour. Cysteine at a final concentration of 30 mM was added to quench the alkylation reaction at 25 °C for another half hour. Each sample was digested with Lys-C (at an enzyme-to-substrate ratio of 1:100, w/w) for 3 h at 37 °C. The protein solution was diluted with 100 mM NH_4_HCO_3_ (pH 8.0) and then digested with trypsin (at an enzyme-to-substrate ratio of 1:50, w/w) at 37 °C for 16 h. Sep-Pak C18 cartridges (Waters, Milford, MA, USA) were used for peptide desalting.

### Tandem mass tag (TMT) labeling

TMT labeling was performed with TMT Mass Tagging Kits (Thermo Fisher Scientiﬁc, San Jose, CA, USA). Tags 126, 127, 130, and 131 were used for DMSO, nifedipine, nortriptyline, and metformin samples, respectively. The labeling efficiency of TMT was verified with an EASY-nLC 1,000 system coupled to an Orbitrap Fusion mass spectrometer (Thermo Fisher Scientiﬁc, San Jose, CA, USA). After labeling assessment, the TMT-tagged peptides from each sample were pooled and desalted with Sep-Pak C18 cartridges (Waters, Milford, MA, USA) before fractionation.

### HPLC fractionation

High-pH reversed-phase HPLC with a Waters XBridge Prep C18 column (5 µm particles, 4.6 × 250 mm) was used to separate the tryptic peptides^23,24^ Mobile phase A (pH = 10) consisted of 2% ACN and ammonium hydroxide solution, and mobile phase B consisted of 98% ACN and 2% mobile phase A. The separation was accomplished at a mobile phase flow rate of 1 mL/min with the following linear gradient: 0% to 5% B for 2 min, 5% to 12% B for 8 min, 12% to 33% B for 57 min, and 33% to 95% B for 2 min. The peptides were finally combined into 20 fractions and vacuum-dried for further experiments.

### Nano-HPLC–MS/MS analysis

Peptide samples were analyzed with nano-HPLC-MS/MS^22,25^. Peptides were dissolved in solvent A (0.1% FA in 2% ACN) and directly loaded onto a homemade reversed-phase C18 analytical column (21 cm length with a 75 µm inner diameter and packed with 3 µm-sized particles) with a linear gradient of 6%–30% solvent B (0.1% FA in 90% ACN) for 57 min, 30%–45% solvent B for 4 min at a constant flow rate of 300 nL/min, and 45%–80% solvent B for 4 min on an EASY-nLC 1,000 system. The eluted peptides were ionized and sprayed into a Q Exactive instrument (Thermo Fisher Scientific, Waltham, MA, USA) *via* a nanoelectrospray source. Peptides with *m/z* ranging from 350–1,500 were analyzed in the Orbitrap at a resolution of 70,000 at *m/z* 200. The automatic gain control target was set to 1 × 10^6^, and the maximum ion injection time was 60 ms. The 16 most intense ions were isolated and sequentially subjected to fragmentation *via* higher collision dissociation with a normalized collision energy of 30%. Then, the ion fragments were analyzed in the Orbitrap at a resolution of 17,500 at *m/z* 200. The isolation window was 2 *m/z*. The dynamic exclusion duration was set to 60 s, and the charge exclusion was set as 1+ and ≥5+.

### Proteomic database search

The MS/MS spectra were analyzed with MaxQuant (v1.5.3.8)^26^ and the built-in Andromeda search engine against the UniProt human sequence database (updated on 8/27/2018, 95128 sequences), thus enabling searches for the reversed versions of all sequences and contaminants. For the TMT labeling samples, TMT-labeled N-termini and lysine residues, and cysteine carbamidomethylation were included as fixed modifications. In addition, methionine oxidation and protein N-terminal acetylation were set as variable modifications. Trypsin/P was chosen as the digestion enzyme, and 2 missed cleavages were allowed. The false discovery rate cutoff used for both peptides and proteins was 0.01 (1%) with the decoy database. The precursor intensity fraction was set as 0.75 to minimize the influence of the coeluting peptides on the quantification.

### Determination of drug effects *in vivo* with a xenograft model

Seven-week-old male athymic nude mice were purchased from Shanghai Laboratory Animal Center. All mice were maintained in a specific-pathogen-free facility, and all related procedures complied with the Guide for the Care and Use of Laboratory Animals and were approved by the institutional biomedical research ethics committee of Shanghai Institute of Nutrition and Health Sciences, Chinese Academy of Sciences (approval No. SIBS-2018-QJ-1). The xenografts were generated by subcutaneous injection of 2 × 10^6^ DU145 cells resuspended in 100 µL sterile PBS. When the tumors reached a volume of approximately 50 mm^3^, the mice were randomized to groups treated with vehicle (saline solution), or a single dose of 5-FU (50 mg/kg), nortriptyline (30 mg/kg) or nifedipine (50 mg/kg) intraperitoneally. The 5-FU was dissolved in saline solution, nortriptyline was dissolved in 0.1% ethanol, and nifedipine was dissolved in 0.25% CMC Na and 0.05% Tween 80. The 5-FU was administered twice per week, whereas nortriptyline and nifedipine were administered every day. The tumor volume was measured with calipers every 3 days, and the weights of the mice were also monitored. The tumor volume was calculated with the formula *V = L × S*^2^ × 0.52, in which *L* represents the long axis of the tumor, and *S* represents the short axis.

## Results

### Model construction

**Figure 1:**
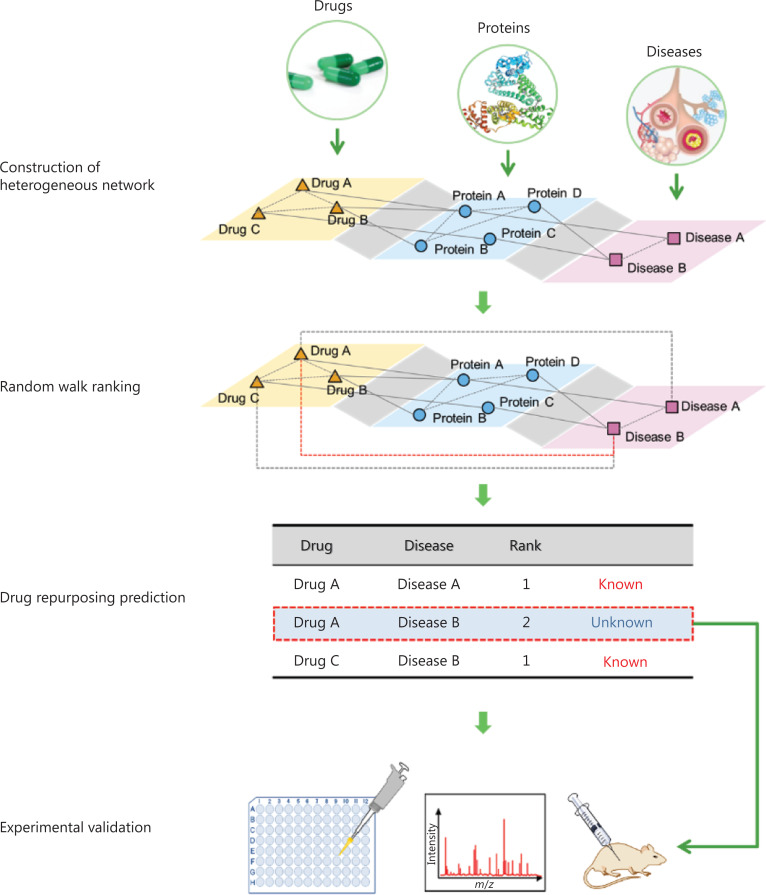
Drug repurposing workflow. A multilayer drug-protein-disease heterogeneous network was constructed on the basis of public databases. Then, a random walk method was implemented to identify potential drug-disease relationships. Novel candidates were validated with *in vitro* and *in vivo* experimental tests.

The occurrence and development of many human diseases are associated with certain genes or proteins, some of which may be potential drug targets. Thus, we hypothesized that network analysis based on a heterogeneous multilayer network (including multiple types of relationships among drugs, genes, proteins, and diseases) might uncover new potential therapeutic effects of existing drugs. In this work, we analyzed drugs and diseases comprehensively in a six-layer drug-protein-disease heterogeneous network. We applied a network-based random walk with restart algorithm^8,13^ to rank the diseases associated with each drug: each disease was associated with a probability that measured the relationship with the given drug. A disease with a high ranking for a particular drug might have a high probability of being treated with the drug. The general workflow is shown in **Figure 1**.

Because of concerns about bias and the effects of prior knowledge on the direct links between drugs and diseases, we applied 2 strategies in this work. In the first strategy, by excluding the drug-disease direct relationship network, we constructed a large-scale drug-protein-disease network by integrating only 5 networks: the drug-target interaction network, protein-protein interaction network, disease-gene relationship network, drug-drug similarity network, and disease-disease similarity network. The redundant nodes were removed. Finally, a heterogeneous network containing 1,419 drugs, 6,942 proteins and 4,096 diseases was retained. Then, a network-based random walk with restart on the heterogeneous network was used to infer the relationships between the drugs and the diseases. We started a random walk from each drug and extracted the prioritization of diseases when the model was convergent. A disease ranking at the top of the list should have a higher probability of being effectively treated with the given drug. In the second strategy, we additionally integrated the direct drug-disease relationship network determined with the KEGG DRUG and KEGG DISEASE databases^27^ as prior knowledge into the initial network. A network propagation algorithm was also used to perform the prioritization.

### Performance evaluation of the repurposing model

To evaluate the performance of the model without prior drug-disease knowledge, we extracted the experimentally verified drug-disease relationships by integrating information from the KEGG DRUG and KEGG DISEASE databases for cross-validation. A total of 997 relationships between 377 drugs and 308 diseases were retained as the independent validation set. Among these, 38% of drugs were found to treat more than one disease.

**Figure 2:**
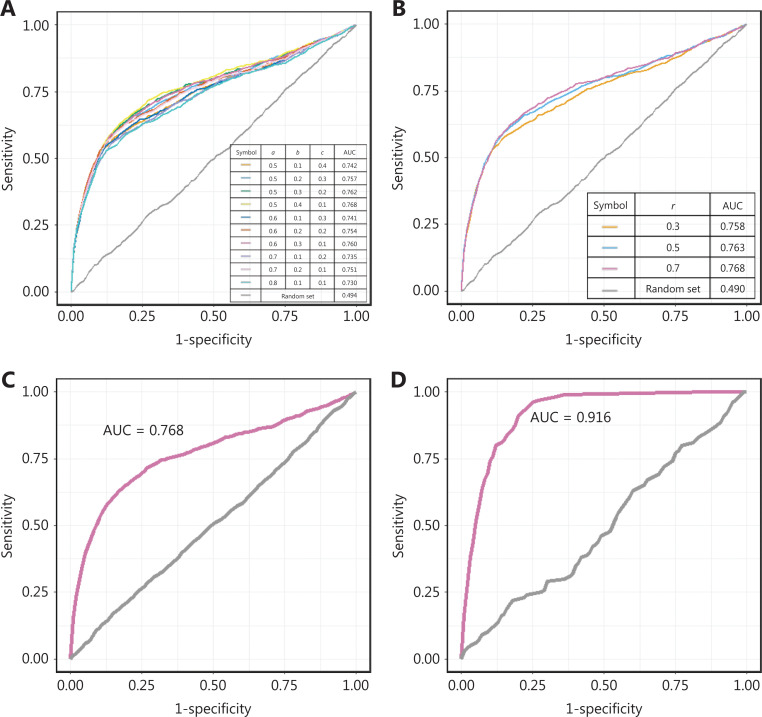
Performance evaluation of the repurposing model. (A–B) The ROC curves of the validation set with the changes in the weighting parameters and restart probability. (C–D) The ROC curves of the model when prior knowledge of the drug-disease links was excluded and included.

We compared the model performance under different conditions to optimize the parameter combinations. There were 4 parameters in our model: the restart probability *r*, and the weighting parameters of the disease, the target protein, and the drug (*a*, *b* and *c*, whose sum is equal to 1), which control the effects of the disease, protein, and drug nodes, respectively, on both the initial and transmission process. We compared the model performance in terms of the AUC value under different weighting parameters and restart probabilities. As demonstrated in **Figure 2A and 2B**, the model was robust to the selection of the parameters. A refined parameter set (*a* = 0.5, *b* = 0.4, *c* = 0.1, and *r* = 0.7) was chosen for the subsequent analysis, because it resulted in a slightly higher AUC value (**Figure 2C**). The parameter optimization process is clarified in **Supplementary materials and methods**.

In previous work, known drug-disease relationships were directly integrated into the heterogeneous network^11,13^, but their effects had not been evaluated. Here, to measure the effects of prior knowledge, we further incorporated the known drug-disease relationships into the heterogeneous network, which was defined as the “full model.” The known drug-disease relationships are listed in **Supplementary Table S1**. Ten-fold cross-validation was also used to measure the full model performance.

The AUC value of the model even without prior knowledge of drug-disease links was 0.768, whereas the full model performance reached 0.916 with an accuracy of 0.86 by incorporating prior knowledge (**Figure 2D**). Prior knowledge thus significantly affected performance (two-sided t test, *P* value < 1e-10). The known drug-disease relationships improved the connectivity of the network and shortened the distances of potential drug-disease pairs in terms of topology. We expect that the model performance will further improve if additional validated drug-disease prior knowledge is integrated in the future. On the basis of its improved performance, we chose the full model for subsequent analysis.

To further evaluate the efficiency and reliability of the drug-disease prioritization in our model, we selected 4 well-established drugs—sirolimus, metformin, itraconazole and risperidone—and investigated the top-ranked diseases for repurposing by literature mining.

We first extracted the top 20-ranked diseases (top group) for each drug and randomly selected 20 diseases (random group) for the comparison. We searched for the drug-disease relationships in the KEGG^27^, DrugBank^17^ and ClinicalTrials.gov^28,29^ databases to determine whether the relationships had been reported in any of these 3 databases. DrugBank and ClincalTrials.gov contain information about most drug-disease relationships revealed by studies in progress or completed clinical trials. For the remaining unreported relationships, we performed manual PubMed literature mining of the supporting literature. The drug-disease relationships not found in databases or the literature were considered to be unavailable.

**Figure 3:**
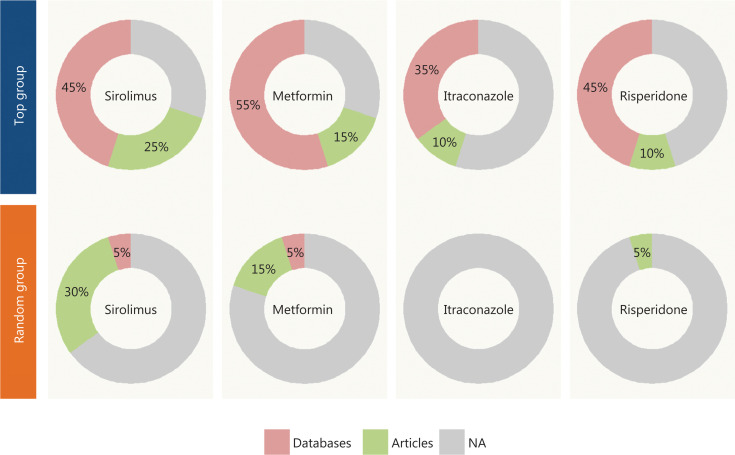
The statistics of the validation information for the top group and the random group. The top 20 diseases for each drug were extracted as the top group and evaluated against 3 databases (KEGG, DrugBank, and ClinicalTrials.gov) as well as PubMed to determine whether the drug-disease relationships had been validated. A random group of 20 diseases was also extracted for comparison.

**Figure 3** shows that approximately 60% of drug-disease relationships associated with repurposing in the top group had been validated or studied in clinical trials, whereas the average supporting ratio was only 15% in the random group, a value significantly lower than that in the top group (one-sided Fisher’s exact test, *P* < 0.05). The details are presented in **Supplementary Tables S2 and S3**.

### Drug sensitivity validation in cancer cell lines

**Figure 4:**
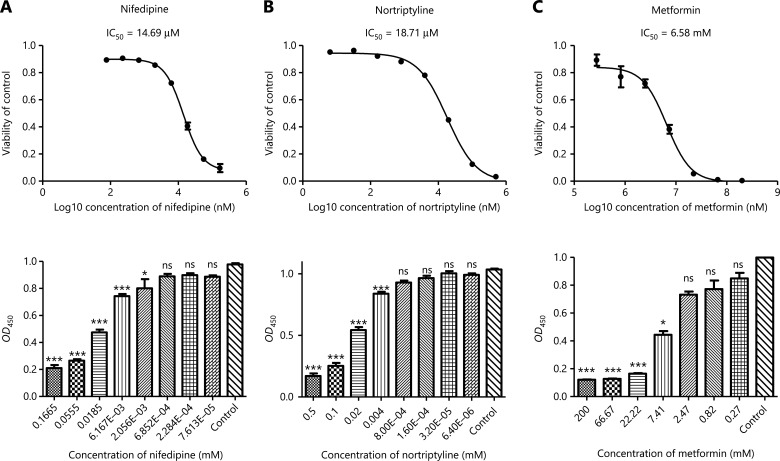
Experimental validation of cancer cell growth inhibition caused by nifedipine, nortriptyline, and metformin in LNCaP cells. (A–C) Nifedipine, nortriptyline, and metformin, compared with DMSO, produced dose-dependent antiproliferative effects in LNCaP cells (*n* = 3). Nifedipine and nortriptyline both produced much lower IC_50_ values than metformin, a repurposed drug reported to have antitumor activity. Cell viability was measured with cell counting kit-8 (CCK8) assays, and *OD*_450_ represented the absorbance (optical density, *OD*) read at a wavelength of 450 nm. Data are presented as the mean ± SEM. Statistical significance was calculated with the Kruskal-Wallis test and Dunn’s test (multiple comparisons among treatment groups and controls). One asterisk indicates *P* < 0.05, 2 asterisks indicate *P* < 0.01, and 3 asterisks indicate *P* < 0.001. NS represents no statistical significance.

To further validate the utility of the novel drug-disease relationships predicted by our model, we selected 5 drugs that had types of cancer in their top-ranked disease list for repurposing and conducted drug sensitivity validation in cell lines. Five drugs (dextromethorphan, tetracycline, nifedipine, atorvastatin, and nortriptyline) were chosen because of their widespread availability and clinical use, as well as an absence of any anticancer effects reported in the literature. Dextromethorphan is widely used in the treatment of cough. Tetracycline, a broad-spectrum antibiotic, shows efficacy in treating bacterial infections. Nifedipine, as a prototypical calcium channel antagonist, is on the World Health Organization List of Essential Medicines^30^. Atorvastatin is a lipid-lowering agent commonly used for the prevention of cardiovascular disease. Nortriptyline, a tricyclic antidepressant, has superior pharmacological properties to those of other tricyclics as a psychotropic drug with improved effects and decreased adverse effects and toxicity^31^. Moreover, metformin, in our top ranked list, was chosen as a positive control for the selected drugs (**Figure 4C and Supplementary Figure S1**) because it has already been repurposed, on the basis of its therapeutic effects on diverse cancers, including breast cancer^32^, prostate cancer^33^, and gastric cancer^34^.

To select the cell line models, we mainly focused on the cancer types ranked in the top 3% in our model. The selected relationships involved dextromethorphan (breast cancer, top 2.64%), tetracycline (prostate cancer, top 0.15%; gastric cancer, top 0.20%), nifedipine (prostate cancer, top 1.00%; gastric cancer, top 1.25%), atorvastatin (gastric cancer, top 0.39%), and nortriptyline (prostate cancer, 0.02%). Therefore, the MCF-7 and MDA231-LM2-4175 human breast cancer cell lines were selected for studying breast cancer; the LNCaP and DU145 human prostate cancer cell lines were selected for studying prostate cancer; and the MGC-803 cell line was selected for studying gastric cancer. To evaluate the dosage effects of these drugs, we used CCK-8 assays to detect cell viability.

The half maximal inhibition of cell viability (IC_50_) values for the cell line studies are presented in **Table 1**. Tetracycline, nifedipine, and nortriptyline produced dose-dependent antiproliferative effects in both LNCaP (**Figure 4A and 4B**) and DU145 cells. Dextromethorphan treatment, compared with DMSO treatment, resulted in a remarkable dose-dependent decrease in survival of both MCF-7 and MDA231-LM2-4175 cells. Tetracycline, nifedipine, and atorvastatin showed toxicity in the MGC-803 cell line. Hence, these results indicated that the IC_50_ levels of the above drugs were nearly 10 to 1,000 times lower than those of metformin (approximately 5–30 mM), thus suggesting that these drugs may have potential antitumor activity, at least at the cellular level. In addition, we further validated our repurposing model by using cancer cell lines (breast cancer cell lines, prostate cancer cell lines, and a gastric cancer cell line). The model predicted low-ranking drugs (chlorpropamide, tolazoline, tiaprofenic acid, and decamethonium). We hypothesized that these drugs should not be toxic even at a high concentration of 100 µM. As we predicted, these drugs had no significant inhibitory effects on the respective cancer cell lines (**Supplementary Figure S2**).

**Table 1:** Cytotoxicity of dextromethorphan, tetracycline, nifedipine, atorvastatin, and nortriptyline against the respective cancer cell lines (*n* = 3)

Drug	Cancer type	Rank (%)	Cell line	IC_50_ (µM)
Dextromethorphan HBr monohydrate	Breast cancer	Top 2.64	MCF-7	71.52 ± 1.84
			MDA231-LM2-4175	95.83 ± 28.03
Tetracycline hydrochloride	Prostate cancer	Top 0.15	LNCaP	13.79 ± 4.50
			DU145	35.35 ± 14.37
	Gastric cancer	Top 0.20	MGC-803	46.86 ± 11.65
Nifedipine	Prostate cancer	Top 1.00	LNCaP	14.69 ± 2.89
			DU145	26.66 ± 19.26
	Gastric cancer	Top 1.25	MGC-803	20.73 ± 10.10
Atorvastatin calcium	Gastric cancer	Top 0.39	MGC-803	4.60 ± 1.69
Nortriptyline hydrochloride	Prostate cancer	Top 0.02	LNCaP	18.71 ± 3.48
			DU145	23.81 ± 7.51
Metformin	Breast cancer	Top 0.15	MCF-7	11.11 × 10^3^–33.33 × 10^3^
			MDA231-LM2-4175	11.11 × 10^3^–33.33 × 10^3^
	Prostate cancer	Top 0.17	DU145	3.7 × 10^3^–11.11 × 10^3^
			LNCaP	~6.58 × 10^3^
	Gastric cancer	Top 0.02	MGC-803	3.7 × 10^3^–11.11 × 10^3^

IC_50_ represents the mean ± standard deviation.

Together, both the literature and the experimental validation indicated that the drug repurposing ranking based on our model was well validated by the *in vitro* models.

### Proteomic characterization of nifedipine/nortriptyline in prostate cancer

**Figure 5:**
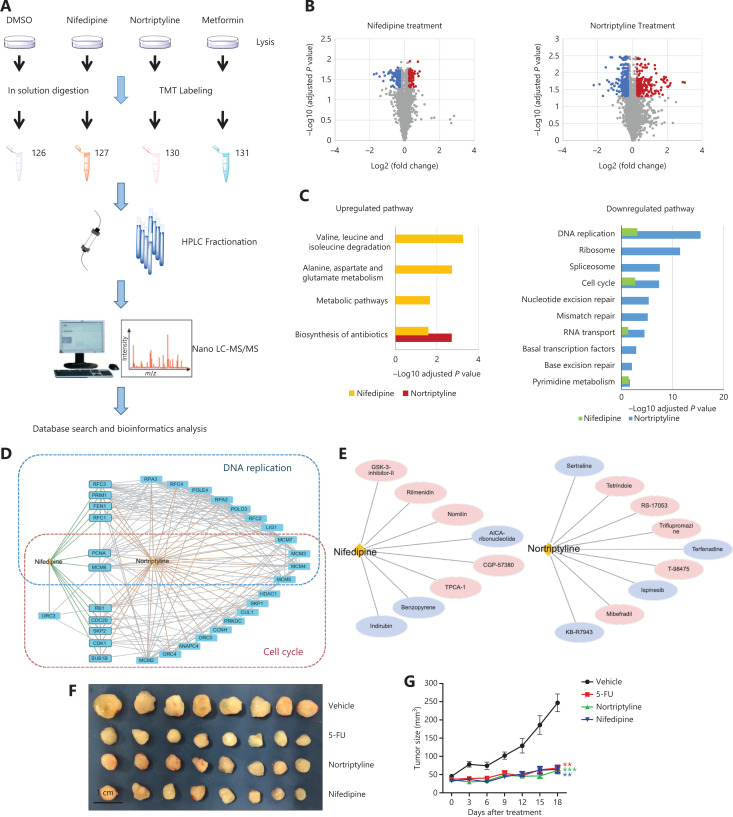
Nifedipine and nortriptyline induced changes in global protein expression in LNCaP cells and inhibited the growth of prostate tumors in a xenograft model. (A) The workflow of the TMT-based quantitative proteomic profiling of LNCaP cells with treatment of DMSO, 70 μM nifedipine, 70 μM nortriptyline, and 70 μM metformin. (B) A volcano plot was used to relate the fold change to the adjusted *P* value. Red dots represent proteins with significant differences and fold changes >1.2, whereas blue dots represent proteins with significant differences and fold changes <0.83. (C) KEGG pathway analysis of up- and downregulated proteins in response to nifedipine and nortriptyline treatment. (D) Protein–protein interaction network of downregulated proteins in 2 important pathways (DNA replication and cell cycle) upon nifedipine/nortriptyline treatment. Interaction networks are listed with gene names according to the STRING database. (E) Drug compound similarity in terms of the gene expression profile from CMAP. The compounds reported to have effects on prostate cancer are highlighted in purple. (F) Image of tumors from mice treated with vehicle (saline solution), or a single dose of 5-FU (50 mg/kg), nortriptyline (30 mg/kg), or nifedipine (50 mg/kg) intraperitoneally (*n* = 8 per group). (G) Tumor growth curve of mice. Data are presented as the mean ± SEM, and statistical significance was calculated with the Kruskal-Wallis test and Dunn’s test (multiple comparisons among treatment groups and controls). One asterisk indicates *P* < 0.05, 2 asterisks indicate *P* < 0.01, and 3 asterisks indicate *P* < 0.001.

The cell line experiments above supported the druggable effects of several predicted drug-disease pairs. To better understand how these drugs affect diseases according to these relationships, we chose 2 candidates, nifedipine and nortriptyline, and used them to treat LNCaP cells for a quantitative proteomics study. TMT labeling and mass spectrometry analysis were used to identify changes in protein expression in response to nifedipine and nortriptyline treatment, respectively (**Figure 5A**). Cells were treated with 70 µM nifedipine/nortriptyline (five times the IC_50_) for 24 h. Cell lysates were extracted, and the proteins were quantified by mass spectrometry after trypsin digestion and TMT labeling. A total of 6,334 proteins were quantified with confidence in our proteomics studies. After filtering for proteins observed in 2 biological replicates, 5,037 proteins with their relative abundances were used in the subsequent analysis. Correlation analysis was performed to evaluate the data quality (**Supplementary Figure S3**). For nifedipine, 635 proteins (12.61%) showed significant differences between the treatment and control groups (permutation false discovery rate <0.05), including 89 upregulated proteins (fold change >1.2) and 147 downregulated proteins (fold change <0.83). In the nortriptyline-treated cells, 1,409 proteins (27.97%) exhibited significant differences, including 275 upregulated proteins and 478 downregulated proteins (**Figure 5B**). No significantly differentially expressed proteins were found in the metformin group, thus indicating that the drug at a low concentration of 70 µM induced little effect in terms of changes in protein expression.

To further explore the pathways perturbed by nifedipine and nortriptyline, we performed pathway enrichment analysis. The results (**Figure 5C**) showed that several important pathways were affected by both drugs, such as DNA replication, the cell cycle, and RNA transport pathways. Most of these pathways are closely associated with cancer development and progression, thus indicating that these 2 drugs may affect similar cellular pathways involved in the induction of growth inhibition. Additionally, multiple proteins, such as PCNA, MCM6, MCM3, and MCM4, involved in the pathways of DNA replication and the cell cycle, were significantly downregulated in both the nifedipine and nortriptyline groups (**Figure 5D**), and consequently may play central roles in nifedipine- and nortriptyline-induced inhibition of cell proliferation in prostate cancer cells. Meanwhile, nifedipine and nortriptyline selectively affected other pathways. For nifedipine, the selectively up- and downregulated proteins were mainly components involved in metabolic processes, such as metabolic pathways and alanine, aspartate, and glutamate metabolism, thus suggesting that nifedipine affects cell viability partly by regulating energy metabolism. In contrast, proteins downregulated by nortriptyline were associated with mismatch repair, nucleotide excision repair, and basal transcription factors. The proteins RPA3 and POLD3, which are involved in these processes, were significantly downregulated in nortriptyline-treated cells. The differences in the enriched pathways implied that the 2 drugs also have distinct mechanisms of decreasing cell viability.

In addition to the evidence at the proteome level, we also searched for compounds producing similar perturbation-driven gene expression profiles to those induced by nifedipine and nortriptyline. By comparing nifedipine and nortriptyline with other compounds in terms of the gene expression profile in Connectivity Map (CMAP), we identified 8 compounds showing high similarity to nifedipine, and 9 compounds showed high similarity to nortriptyline (connectivity score >99) in their gene expression profiles (**Figure 5E**). Twelve of these compounds have been reported to show antitumor effects through different mechanisms, thus providing additional clues as to how nifedipine and nortriptyline may affect cancer cells at the gene expression level. The details on the compounds and their anticancer activities are shown in **Supplementary Table S4**. The compound indirubin shows high similarity to nifedipine in terms of perturbing gene expression in human cancer cell lines, and indirubin has been shown to inhibit cyclin-dependent kinases, thus resulting in cell cycle arrest and the inhibition of cell proliferation^35,36^. The high similarity between indirubin and nifedipine in regulating gene expression in human cancer cell lines suggests that nifedipine affects cell survival through similar mechanisms. In addition, KB-R7943, a sodium/calcium exchange inhibitor, is highly similar to nortriptyline, according to the CMAP. Recently, KB-R7943 has been found to activate the JNK signaling pathway and block autophagic flux, thereby promoting cell death in prostate cancer^37^. The data also suggested that nortriptyline might affect cell survival through similar mechanisms.

### Nifedipine and nortriptyline inhibit the growth of prostate tumors in a xenograft model

The *in vitro* experiments suggested that nifedipine and nortriptyline have effects on prostate cancer. To further investigate these anticancer effects *in vivo*, we used a xenograft model in male athymic nude mice. DU145 cells were subcutaneously injected into 32 mice. When the tumors reached a volume of approximately 50 mm^3^, the mice were randomized to groups treated with vehicle (saline solution), or a single dose of 5-fluorouracil (5-FU, 50 mg/kg), nortriptyline (30 mg/kg), or nifedipine (50 mg/kg) intraperitoneally. In line with our expectations, 5-FU treatment decreased the growth of prostate cancer cells. In addition, nifedipine and nortriptyline, compared with the vehicle, significantly inhibited the growth and proliferation of tumors (**Figure 5F and 5G**). The average body weight changes in the vehicle group, 5-FU group, nortriptyline group, and nifedipine group are shown in **Supplementary Figure S4**. No differences in average body weights were observed in treated mice. Collectively, these findings demonstrated that nifedipine/nortriptyline treatment showed *in vivo* efficacy without causing apparent body weight changes.

### Database construction

We have uploaded our drug-repurposing results to a public database named PAD (The Predictive Database for Drug Repurposing) (URL: http://lilab.life.sjtu.edu.cn:8080/pad/index.html) to enable the discovery of new potential efficacies of known drugs (**Supplementary Figure S5**). PAD contains information on the relationships between 1,419 drugs and 4,096 diseases. Clinical information from the KEGG, DrugBank, and ClinicalTrials.gov is provided to assist researchers in prefiltering known drug-disease relationships. The Human Metabolome Database (hmdb)^38^ is integrated to indicate whether the drug is endogenous to the human body. Three data searching methods are available in PAD: searching by drug, disease, or drug-disease pair.

## Discussion

Multiple studies have demonstrated the utility of drug repurposing in drug discovery and development. Although different computational approaches have been proposed to discover potential drug-disease relationships, efficient methods or resources for this purpose remain limited. In this study, we comprehensively included many drugs and diseases in a six-layer heterogeneous network and applied a global random walk algorithm for drug repurposing. The model evaluation revealed that the full model, which integrated known direct drug-disease relationships, had a higher AUC value and improved accuracy than the model without prior knowledge. Several newly predicted drug-disease relationships were chosen for validation through *in vitro* drug sensitivity experiments in cancer cell lines and *in vivo* detection of antitumor effects in a xenograft model. To characterize the mechanisms involved in the new drug-disease relationships, we performed a TMT-based quantitative proteomics experiment as well as analysis of compound perturbation-driven gene expression profiles by using a public database. The results expanded the basis for evaluating the pharmacological effects of repurposed drugs, thus demonstrating the reliability of our predictions. Finally, a predictive database for drug repurposing with a user-friendly interface was built on the basis of our results.

We subsequently evaluated our model with different parameters and initial matrices. Comprehensive comparisons between our model and previous models were not conducted, owing to the lack of availability of the source code or web tools^11^. Moreover, other methods are based on different databases, thus potentially resulting in terminology issues, such as a lack of term matching. Researchers may address this problem by implementing manual checking; however, this correction strategy may easily produce artifacts^11^. Therefore, we used MeSH terms as the disease identifiers in our model and database.

Our proteomics study on the cell lines treated with nifedipine and nortriptyline suggested corresponding differentially expressed proteins and up- or down-regulated pathways. For example, the cytotoxicity of nortriptyline might be due to its effects on cell cycle arrest (**Supplementary Figure S6**). Previous reports have shown that the CDK4 and CDK6/cyclin D complexes contribute to the G1-S transition by phosphorylating the retinoblastoma (Rb) protein^39,40^. After the phosphorylation of Rb, E2F is separated from the Rb/E2F complex, thus activating the expression of genes that are necessary for S phase transition^41^. Our data showed that the expression of CDK4 did not exhibit significant changes, but the expression of Rb was downregulated by nortriptyline, thus potentially affecting the Rb/E2F complex and consequently inhibiting the expression of E2F target genes. Simultaneously, nortriptyline significantly downregulated CDK1, which also notably participates in the regulation of the eukaryotic cell cycle^42–44^. Moreover, we searched the reported targets of nifedipine and nortriptyline in the DrugBank database and previous publications. We found that the targets of nifedipine, such as voltage-dependent L-type calcium channel subunit alpha-1C (CACNA1C), subunit alpha-1D (CACNA1D) and beta-2 (CACNB2), have important links to cancer development and progression^45–49^. Furthermore, one target of nortriptyline, sodium-dependent noradrenaline transporter (SLC6A2), also affects important pathways in cancer^50^. These clues at the proteomics level and the reported drug targets might contribute to nifedipine/nortriptyline-induced antitumor effects but must be further validated in additional independent experiments.

Although our full model achieved good performance for drug repurposing, integration of more biological evidence, such as drug adverse effect similarities and pathway correlations, could further improve the model’s performance. Moreover, model performance would probably be enhanced if additional validated prior knowledge were integrated.

## Conclusions

In summary, our model and the online resource PAD offer a systematic approach for performing reliable drug repurposing prediction to discover potential drug-disease relationships, which may accelerate drug research and therapeutic development.
