# Supplementary material for: Drug repurposing for cancer treatment through global propagation with a greedy algorithm in a multilayer network
Source: Cancer Biol Med. 2022 Jan 15;19(1):74–89. doi: 10.20892/j.issn.2095-3941.2020.0218 (PMC8762999; doi:10.20892/j.issn.2095-3941.2020.0218)
Supplement: Supplementary file 1 [file cbm-19-074-s001.pdf]

Supplementary materials

Supplementary materials and methods

Random walk in the heterogeneous network

1. Determination of initial probability p_0

In the initial probability p_0, probability 1 was assigned to the seed nodes, and probability 0 was assigned to other vertices, forming the drug network h_0 and the protein network v_0. Given that we added disease nodes, the initial probability of disease network u_0 is a zero vector containing no seed nodes. Hence, the initial probability of the heterogeneous network can be represented as:

$$p_0 = \begin{bmatrix} au_0 \\ bv_0 \\ ch_0 \end{bmatrix}$$

2. Construction of transition matrix *M*

In the transition matrix *M*, $M_{disease-disease}$, $M_{protein-protein}$ and $M_{drug-drug}$ are inter-transition matrices representing the probability of the transition from one disease/protein/drug to another disease/protein/drug node. $M_{disease-protein}$ is the transition matrix from the disease network to the protein network, whereas $M_{protein-disease}$ is the transition matrix from the protein network to the disease network. Similarly, $M_{protein-drug}$ is the transition matrix from the protein network to the drug network, whereas $M_{drug-protein}$ is the transition matrix from the drug network to the protein network.

The transition probability from vertex disease *i* to protein *j* was defined as:

$$M_{disease-protein}(i,j) = \frac{bS_1(i,j)}{\sum_j S_1(i,j)}$$

The transition probability from vertex protein *i* to disease *j* was defined as:

$$M_{protein-disease}(i,j) = \frac{aS_1(j,i)}{\sum_j S_1(j,i)}$$

The transition probability from vertex drug *i* to protein *j* was defined as:

$$M_{drug-protein}(i,j) = \frac{bS_2(i,j)}{\sum_j S_2(i,j)}$$

The transition probability from vertex protein *i* to drug *j* was defined as:

$$M_{protein-drug}(i,j) = \frac{cS_2(j,i)}{\sum_j S_2(j,i)}$$

The transition probability from vertex protein *i* to protein *j* was defined as:

$$M_{protein-protein}(i,j) = \begin{cases} \frac{bS_3(i,j)}{\sum_j S_3(i,j)} & if \sum_j S_3(i,j) \neq 0 \\ 0 & otherwise \end{cases}$$

The transition probability from vertex drug *i* to drug *j* was defined as:

$$M_{drug-drug}(i,j) = \frac{cS_4(i,j)}{\sum_j S_4(i,j)}$$

The transition probability from vertex disease *i* to disease *j* was defined as:

$$M_{disease-disease}(i,j) = \frac{aS_5(i,j)}{\sum_j S_5(i,j)}$$

In the first strategy, $M_{disease-drug}$ and $M_{drug-disease}$ were set as zero matrices. However, in the second strategy, the transition probability from vertex drug *i* to disease *j* was defined as:

$$M_{drug-disease}(i,j) = \begin{cases} \frac{aS_6(i,j)}{\sum_j S_6(i,j)} & if \sum_j S_6(i,j) \neq 0 \\ 0 & otherwise \end{cases}$$

The transition probability from vertex disease *i* to drug *j* was defined as:

$$M_{disease-protein}(i,j) = \begin{cases} \frac{cS_6(j,i)}{\sum_j S_6(j,i)} & if \sum_j S_6(j,i) \neq 0 \\ 0 & otherwise \end{cases}$$

Then the random walk can be implemented on the heterogeneous network based on the transition matrix *M*.

The parameter optimization process

Random walk differs from many other machine learning algorithms in that it does not have a loss function during the iteration. Consequently, it can measure only the final accuracy by cross validation after computing and ranking. Thus, normal

parameter optimization cannot be directly implemented. In this research, we selected as many different parameter combinations as possible within our computing power and used AUC values to measure which parameters combination were optimal.

Here, the weight of drug network *a* was preferentially given a higher proportion (more than 0.5), according to previous studies[1,2]. The random walk model implemented for drug repurposing has been demonstrated to be robust to the selection of *r*; therefore, only 3 values between 0 and 1 (0.3, 0.5, and 0.7) were chosen to test whether the robustness still functions in our model. The results showed that our model was robust to the selection of *r* (AUC value difference ≤ 0.01). Therefore, we chose $r = 0.7$ because it had the best performance in both previous studies and our research.

Enrichment analysis for differentially expressed proteins

The $\log_2$-transformed value of each reporter ion intensity (corrected) was obtained. The SVA package was applied to remove batch effects (**Supplementary Figure S7**). Then the data were imported into Perseus v1.6.1.3 for statistical analysis. The processed intensities were normalized, and two-tailed *t*-tests were performed as described previously[3]. Proteins meeting significance criteria were subjected to analysis with the Database for Annotation, Visualization and Integrated Discovery (DAVID 6.8) tools with the total human genome information as the background. On the basis of fold change, the proteins with significant differences were classified into 2 data sets: the upregulated data set (fold change >1.2) and downregulated data set (fold change <0.83). Kyoto Encyclopedia of Genes and Genomes (KEGG) pathway analysis was used to investigate the molecular mechanisms. The adjusted *P* value (Benjamini–Hochberg correction) cutoff was 0.05.

Network analysis

Cytoscape (version 3.6.1) software based on the STRING database (version 10.5) was used to analyze protein–protein interactions and the downregulated proteins[4,5]. Interactions with

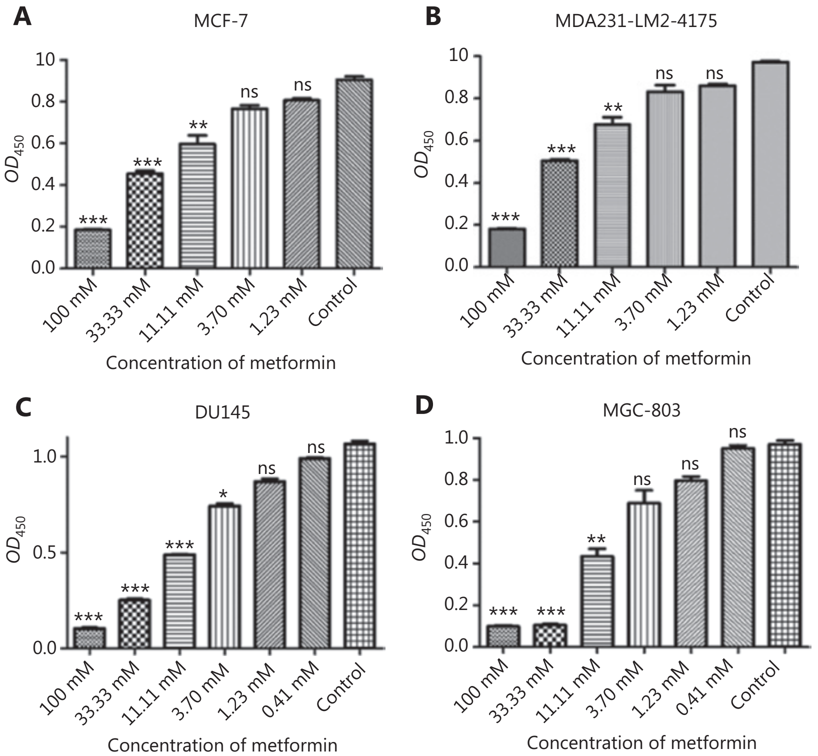

Figure S1 Metformin was chosen as a positive control for the selected drugs in breast cancer cell lines, a prostate cancer cell line, and a gastric cancer cell line. (A)–(D) Compared with DMSO, metformin had dose-dependent antiproliferative effects on breast cancer cell lines, a prostate cancer cell line, and a gastric cancer cell line. Data are presented as the mean ± SEM ($n = 3$). Statistical significance was calculated with the Kruskal-Wallis test and Dunn's test (multiple comparisons among treatment groups and controls). One asterisk indicates $P < 0.05$, 2 asterisks indicate $P < 0.01$, and 3 asterisks indicate $P < 0.001$. NS represents no statistical significance.

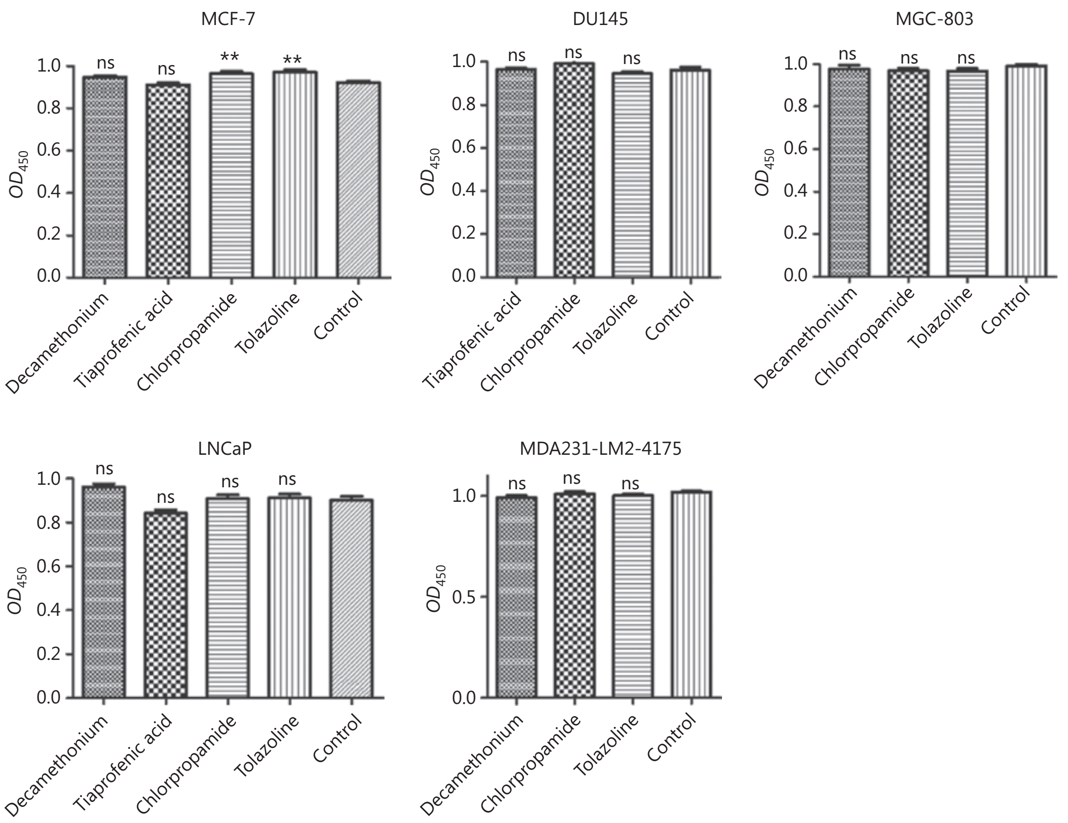

Figure S2 The model predicted low-ranking drugs (chlorpropamide, tolazoline, tiaprofenic acid, and decamethonium) showed no significant inhibitory effects on the respective cancer cell lines, even at a high concentration of 100 μM. The MCF-7 and MDA231-LM2-4175 human breast cancer cell lines were selected for studying breast cancer; the LNCaP and DU145 human prostate cancer cell lines were selected for studying prostate cancer; and the MGC-803 cell line was selected for studying gastric cancer. The model predicted low-ranking drugs (chlorpropamide, tolazoline, tiaprofenic acid, and decamethonium) showed no significant inhibitory effects on the respective cancer cell lines, even at a high concentration of 100 μM. Data are presented as the mean ± SEM (n = 3). Statistical significance was calculated with the Kruskal-Wallis test and Dunn's test (multiple comparisons among treatment groups and controls). One asterisk indicates $P < 0.05$, 2 asterisks indicate $P < 0.01$, and 3 asterisks indicate $P < 0.001$. NS represents no statistical significance.

an interaction score ≥0.7 and active interaction sources from experiments and databases were exported from STRING for Cytoscape analysis.

Analysis of drug-compound similarity in mechanism

Connectivity Map (CMAP) of the Broad Institute Drug Repurposing Hub (https://clue.io), data version 1.1.1.2 and software version 1.1.1.33, was used for further analysis of compound similarity on the basis of gene-expression profiling. CMAP reveals connections among small molecules by measuring the similarity of transcriptional responses to perturbation in different human cell lines[6]. We extracted the items in "Compound" for nifedipine and nortriptyline. The score threshold was set at 99.

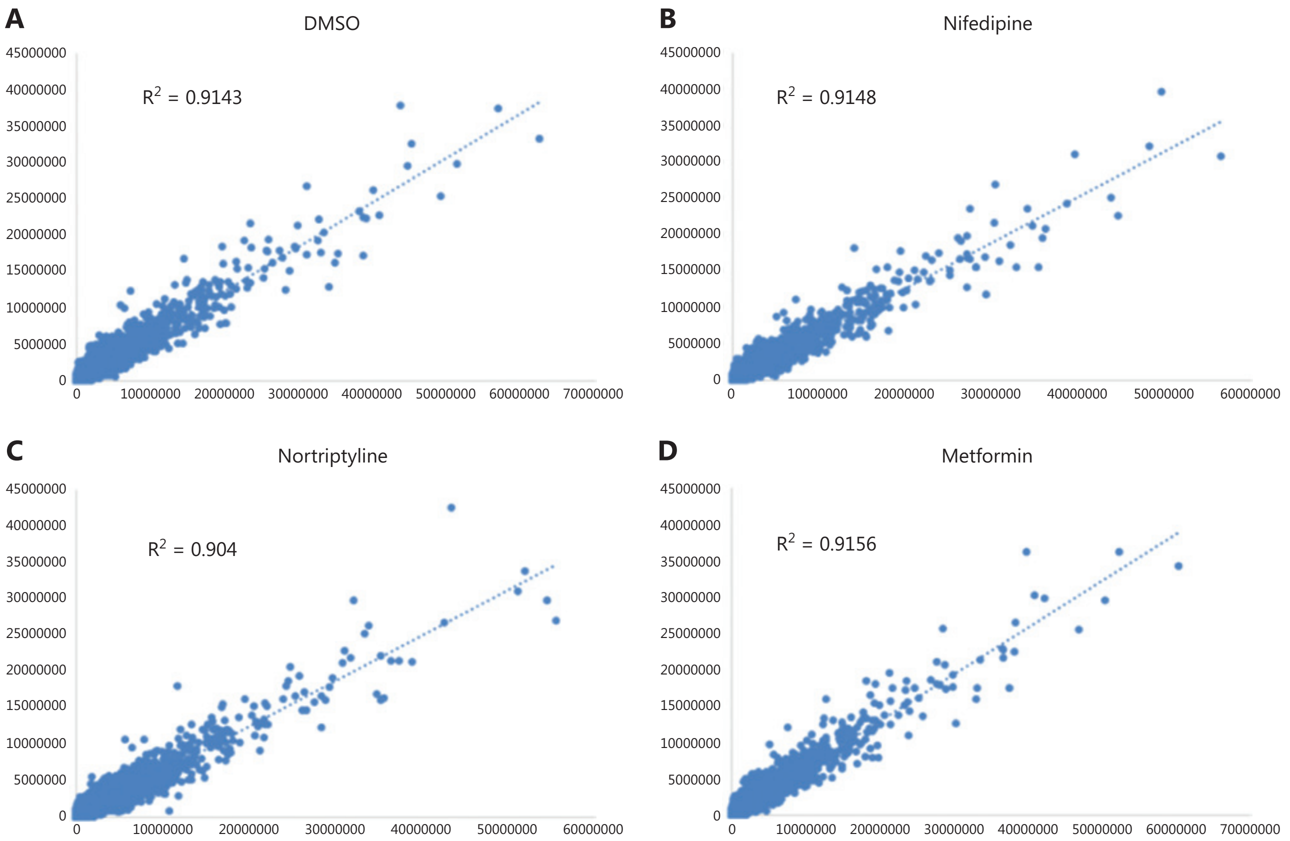

Figure S3 Pearson correlation analysis was performed to evaluate the data quality. Pearson correlation of protein intensities in response to DMSO (A), nifedipine (B), nortriptyline (C), and metformin (D) treatment between 2 biological replicates.

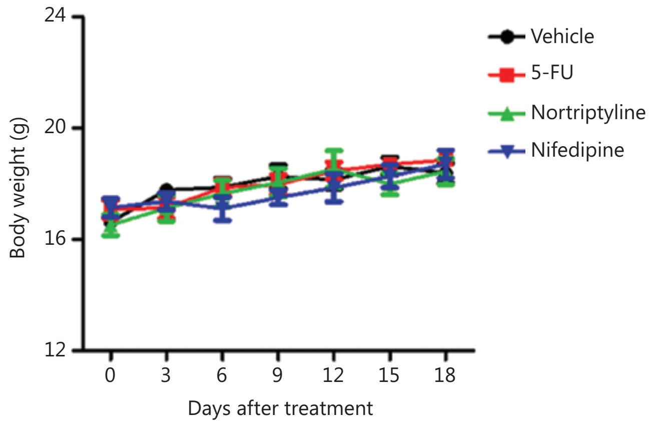

Figure S4 The average body weight changes in mice treated with vehicle (saline solution), or a single dose of 5-FU (50 mg/kg), nortriptyline (30 mg/kg), or nifedipine (50 mg/kg) intraperitoneally (*n* = 8 per group). Data are presented as the mean ± SEM.

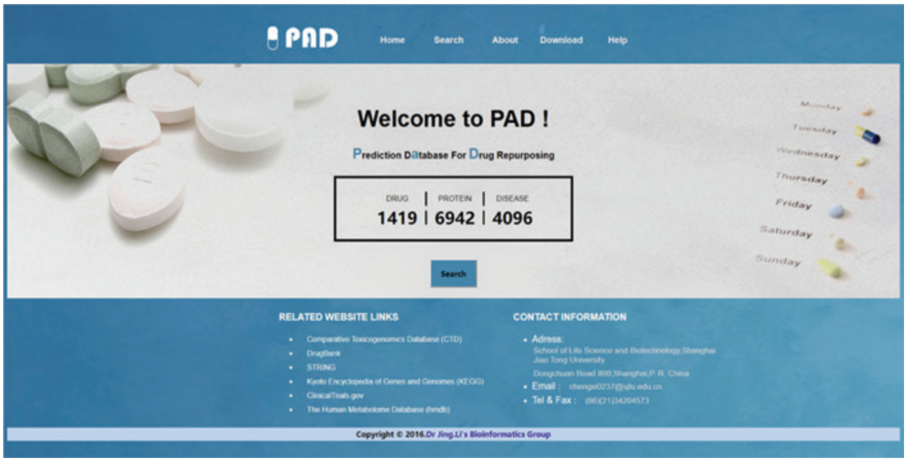

Figure S5 The PAD database interface. The prediction results of the full model were uploaded to the PAD database, which enabled ranking of queries for 1,419 drugs and 4,096 diseases.

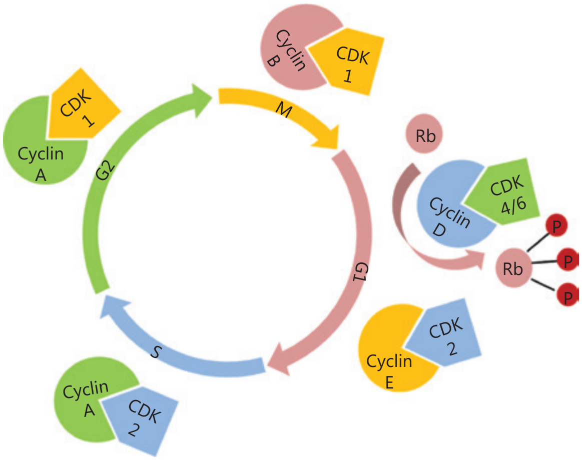

Figure S6 Possible mechanisms of nortriptyline. The cytotoxicity of nortriptyline might be due to its effects on cell cycle progression. Nortriptyline downregulates the expression of Rb, thus potentially affecting the Rb/E2F complex and consequently inhibiting the expression of E2F target genes. In addition, nortriptyline significantly downregulates CDK1. In summary, cell cycle arrest might contribute to the nortriptyline-induced cytotoxicity.

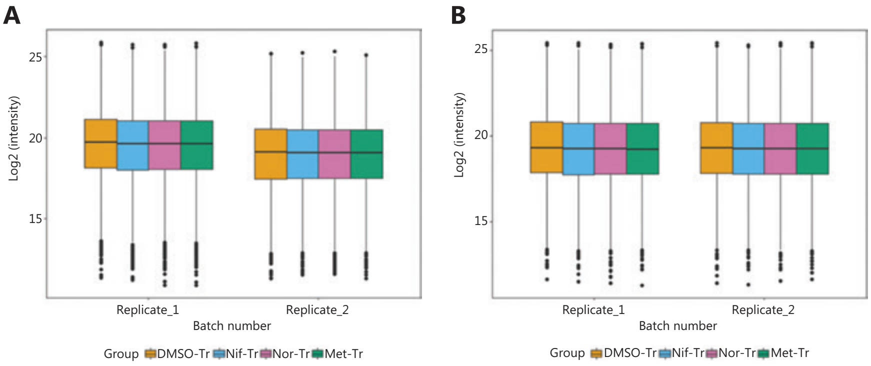

Figure S7 The SVA package was applied to remove batch effects. (A) Distribution of $\log_2$ (intensity) of 4 groups (DMSO, nifedipine, nortriptyline, and metformin) before removal of batch effects. (B) Distribution of $\log_2$ (intensity) of 4 groups (DMSO, nifedipine, nortriptyline, and metformin) after removal of batch effects.

Table S1 The known drug-disease relationships from KEGG

DrugBank ID	Disease MeSH ID
DB00997	D006689
DB00290	D006689
DB00570	D006689
DB00851	D006689
DB00773	D006689
DB00541	D006689
DB00635	D006689
DB00531	D006689
DB01168	D006689
DB00945	D013920
DB01005	D013920
DB00999	D006973
DB00310	D006973
DB00524	D006973
DB00808	D006973
DB00695	D006973
DB00903	D006973
DB00887	D006973
DB00214	D006973
DB00421	D006973
DB00594	D006973
DB00700	D006973
DB01193	D006973
DB00335	D006973
DB00612	D006973
DB01136	D006973
DB00598	D006973
DB00264	D006973
DB01203	D006973
DB01359	D006973
DB00960	D006973
DB00571	D006973
DB00373	D006973
DB00542	D006973
DB01197	D006973
DB00584	D006973

Table S1 Continued

DrugBank ID	Disease MeSH ID
DB00492	D006973
DB00722	D006973
DB00691	D006973
DB00881	D006973
DB00178	D006973
DB00519	D006973
DB00661	D006973
DB00796	D006973
DB00876	D006973
DB01029	D006973
DB00678	D006973
DB01013	D011565
DB00443	D011565
DB00596	D011565
DB00223	D011565
DB02300	D011565
DB00936	D011565
DB00799	D011565
DB00864	D011565
DB00459	D011565
DB00091	D011565
DB00563	D011565
DB00125	C565128
DB00152	D007888
DB00126	D007888
DB00583	D007888
DB00666	D007889
DB00338	D057765
DB00448	D057765
DB00213	D057765
DB00736	D057765
DB01129	D057765
DB00588	D057765
DB01222	D057765
DB00635	D000224
DB00741	D000224

Table S1 Continued

DrugBank ID	Disease MeSH ID
DB01234	D000224
DB00687	D000224
DB00619	D008579
DB01005	D008579
DB00104	D008579
DB00959	D001249
DB00860	D001249
DB00635	D001249
DB00938	D001249
DB00983	D001249
DB01222	D001249
DB01003	D001249
DB00716	D001249
DB00471	D001249
DB00549	D001249
DB00744	D001249
DB00277	D001249
DB00394	D001249
DB00180	D001249
DB00764	D001249
DB00620	D001249
DB01291	D001249
DB00332	D001249
DB00583	C538324
DB00997	D018232
DB00851	D018232
DB01181	D018232
DB00619	D018232
DB01268	D018232
DB00997	D018234
DB00851	D018234
DB01181	D018234
DB00619	D018234
DB01268	D018234
DB00178	C535507
DB00966	C535507

Table S1 Continued

DrugBank ID	Disease MeSH ID
DB00678	C535507
DB00451	D003409
DB00125	C567192
DB00451	C563206
DB00666	D011629
DB00666	C536961
DB01119	D006946
DB00635	D020275
DB01164	D004062
DB02300	D004062
DB00136	D004062
DB01436	D004062
DB00264	D000787
DB00335	D000787
DB00612	D000787
DB00343	D000787
DB00661	D000787
DB00883	D000787
DB01020	D000787
DB00243	D000787
DB01039	D000326
DB00227	D000326
DB01013	D010392
DB00959	D004660
DB00864	D004660
DB00877	D001254
DB00997	D018208
DB00851	D018208
DB01181	D018208
DB00619	D018208
DB01268	D018208
DB00396	C537919
DB00624	C537919
DB00783	C537919
DB00188	D009101
DB01234	D009101

Table S1 Continued

DrugBank ID	Disease MeSH ID
DB01041	D009101
DB00480	D009101
DB00635	D009101
DB00421	D001477
DB00384	D001477
DB00328	D001477
DB00550	C563786
DB00199	C536174
DB00541	D054198
DB00635	D054198
DB00694	D054198
DB00563	D054198
DB00531	D054198
DB00997	D054198
DB01234	D054198
DB00987	D054198
DB01033	D054198
DB00451	D050031
DB00641	D050031
DB00819	D050030
DB01144	D050030
DB00136	D053098
DB02701	C536081
DB00627	C536081
DB00413	D012148
DB00996	D012148
DB00980	D007319
DB00656	D007319
DB00583	C536778
DB00133	C567032
DB00145	C567032
DB00687	D011546
DB00695	D011546
DB01393	D006952
DB01039	D006952
DB00175	D006952

Table S1 Continued

DrugBank ID	Disease MeSH ID
DB01393	D006954
DB01039	D006954
DB00035	D020790
DB00564	D000795
DB00909	D004831
DB00349	D004831
DB00829	D004831
DB00555	D004831
DB00593	D004831
DB01202	D004831
DB00515	D015266
DB00773	D015266
DB00997	D015266
DB00694	D015266
DB00445	D015266
DB01177	D015266
DB00385	D015266
DB00290	D015266
DB00531	D015266
DB00819	C567753
DB00195	C567753
DB01194	C567753
DB00521	C567753
DB00869	C567753
DB00654	C567753
DB01210	C567753
DB01085	C567753
DB00373	C567753
DB00287	C567753
DB00480	C535323
DB00653	C566593
DB00619	D015464
DB01222	D003424
DB00515	D013736
DB00773	D013736
DB00290	D013736

Table S1 Continued

DrugBank ID	Disease MeSH ID
DB01181	D013736
DB00433	D012559
DB00850	D012559
DB00477	D012559
DB00623	D012559
DB00531	D017728
DB00997	D017728
DB00541	D017728
DB00635	D017728
DB00695	D007177
DB00531	D016411
DB00997	D016411
DB00541	D016411
DB00635	D016411
DB01137	D011778
DB01208	D011778
DB00254	D011778
DB01611	D011778
DB01165	D011778
DB00487	D011778
DB01045	D011778
DB00608	D011778
DB00396	D017436
DB00624	D017436
DB00783	D017436
DB00162	D012174
DB00343	D012174
DB00541	D012175
DB00773	D012175
DB00242	D007943
DB00531	D020522
DB00997	D020522
DB00541	D020522
DB00635	D020522
DB00188	D020522
DB00126	D000474

Table S1 Continued

DrugBank ID	Disease MeSH ID
DB00373	D013341
DB00654	D013341
DB00776	D013341
DB01202	D013341
DB01174	D013341
DB00273	D013341
DB01080	D013341
DB00945	D013341
DB00515	D008527
DB00531	D008527
DB00541	D008527
DB01030	D008527
DB00125	C535598
DB00133	C566618
DB00643	D014257
DB00518	D014257
DB00515	D008654
DB00642	D008654
DB00441	D008654
DB01016	D003924
DB00331	D003924
DB00284	D003924
DB01132	D003924
DB00731	D003924
DB00379	D003929
DB00476	D003929
DB00230	D003929
DB01029	D003928
DB00678	D003928
DB00741	D017285
DB00443	D017285
DB06151	D017114
DB00091	D006086
DB00864	D006086
DB00563	D006086
DB00688	D006086

Table S1 Continued

DrugBank ID	Disease MeSH ID
DB00959	D006086
DB00635	D006086
DB00993	D006086
DB00635	D016553
DB01234	D016553
DB00515	D009303
DB00544	D009303
DB01229	D009303
DB01248	D009303
DB00181	D054069
DB00140	D054069
DB00583	D054069
DB00675	D016889
DB00515	D016889
DB00997	D016889
DB01229	D016889
DB00544	D018281
DB00428	D018281
DB00441	D018281
DB00945	D016736
DB00398	D013964
DB01268	D013964
DB00653	C537152
DB00999	D006333
DB00524	D006333
DB00310	D006333
DB00727	D006333
DB01234	C536447
DB00741	D000312
DB01234	D000312
DB00860	D000312
DB00687	D000312
DB00555	D020754
DB01229	D010051
DB01248	D010051
DB00515	D010051

Table S1 Continued

DrugBank ID	Disease MeSH ID
DB00441	D010051
DB01030	D010051
DB00531	D010051
DB00773	D010051
DB00746	D006432
DB01082	D011552
DB00798	D011552
DB03615	D011552
DB00452	D011552
DB00169	D014820
DB00443	D014820
DB01234	D014820
DB00583	C535541
DB00515	D009062
DB00544	D009062
DB01229	D009062
DB01248	D009062
DB00641	D054078
DB00152	D008375
DB00451	C564608
DB00763	D013971
DB00997	D006528
DB00945	D009203
DB00571	D009203
DB00584	D009203
DB00641	D009203
DB00727	D009203
DB00811	D006526
DB00763	D006980
DB01229	D010534
DB00515	D010534
DB00437	D007926
DB00415	D011008
DB01053	D011008
DB00417	D011008
DB00713	D011008

Table S1 Continued

DrugBank ID	Disease MeSH ID
DB01327	D011008
DB00493	D011008
DB01212	D011008
DB00535	D011008
DB00199	D011008
DB01211	D011008
DB00778	D011008
DB00207	D011008
DB01165	D011008
DB00537	D011008
DB00487	D011008
DB00467	D011008
DB01059	D011008
DB00978	D011008
DB01137	D011008
DB00685	D011008
DB00218	D011008
DB01208	D011008
DB00860	D012594
DB00635	D012594
DB00959	D012594
DB00563	D012594
DB00531	D012595
DB00338	D012595
DB00213	D012595
DB01129	D012595
DB00736	D012595
DB00448	D012595
DB01240	D012595
DB01197	D012595
DB00584	D012595
DB00722	D012595
DB00790	D012595
DB00178	D012595
DB00881	D012595
DB00542	D012595

Table S1 Continued

DrugBank ID	Disease MeSH ID
DB01340	D012595
DB00492	D012595
DB00519	D012595
DB01348	D012595
DB00691	D012595
DB00559	D012595
DB00851	D010282
DB00544	D010282
DB00531	D010282
DB00472	D020294
DB00908	D020294
DB01364	D020294
DB01001	D020294
DB00190	D010300
DB01235	D010300
DB00810	D010300
DB00915	D010300
DB00360	D010661
DB00441	D010190
DB00515	D010190
DB01229	D010190
DB00530	D010190
DB00544	D010190
DB00762	D010190
DB01068	D019305
DB01595	D019305
DB00349	D019305
DB00690	D019305
DB00593	D019305
DB01202	D019305
DB01174	D006932
DB00419	D005776
DB00550	C566384
DB00328	D004374
DB01050	D004374
DB00379	D020967

Table S1 Continued

DrugBank ID	Disease MeSH ID
DB00819	D020967
DB00999	D020967
DB00495	D015658
DB00503	D015658
DB01393	D008072
DB01039	D008072
DB01200	D005687
DB00248	D005687
DB00437	C538228
DB01586	D008105
DB00715	D001008
DB01175	D001008
DB00829	D001008
DB01268	D002292
DB00398	D002292
DB00515	D002294
DB00997	D002294
DB00795	D013167
DB00125	D017241
DB00847	D009472
DB00959	D009471
DB00860	D009471
DB00993	D009471
DB00688	D009471
DB02701	D011928
DB00627	D011928
DB00443	D011928
DB01049	D011928
DB03904	C564491
DB01045	D007918
DB00845	D007918
DB00819	D020514
DB01144	D020514
DB00761	D020514
DB00125	C537622
DB00819	D020513

Table S1 Continued

DrugBank ID	Disease MeSH ID
DB01144	D020513
DB00999	D020513
DB00379	D020513
DB01001	D020513
DB00396	D007006
DB00624	D007006
DB00783	D007006
DB01068	D020191
DB00313	D020191
DB00909	D020191
DB01202	D020191
DB00448	D005764
DB00501	D005764
DB01241	D015228
DB00159	D015228
DB00627	D015228
DB00091	D015352
DB00437	C538235
DB00959	D014607
DB00635	D014607
DB00860	D014607
DB00443	D014607
DB00091	D014607
DB00993	D014607
DB00175	D006949
DB00641	D006949
DB01095	D006949
DB01098	D006949
DB00973	D006949
DB01393	D006949
DB01039	D006949
DB00636	D006949
DB01599	D006949
DB00515	D002289
DB00361	D002289
DB00773	D002289

Table S1 Continued

DrugBank ID	Disease MeSH ID
DB01229	D002289
DB00441	D002289
DB00642	D002289
DB00515	D002280
DB00997	D002280
DB01200	C537437
DB01037	C537437
DB00165	C537437
DB00686	D018856
DB01062	D018856
DB00248	D003480
DB00648	D003480
DB01011	D003480
DB01026	D003480
DB00834	D003480
DB00257	D002177
DB01167	D002177
DB01026	D002177
DB01110	D002177
DB00928	D009190
DB01262	D009190
DB00531	D009447
DB00997	D009447
DB00515	D009447
DB00773	D009447
DB00379	D009224
DB00819	D009224
DB01035	D009224
DB01220	D006501
DB00136	D007011
DB01436	D007011
DB00741	D007018
DB01077	D007873
DB00720	D007873
DB00282	D007873
DB00630	D007873

Table S1 Continued

DrugBank ID	Disease MeSH ID
DB01133	D007873
DB00710	D007873
DB00884	D007873
DB00399	D007873
DB00419	D052556
DB04841	C536589
DB00704	D000437
DB00659	D000437
DB00822	D000437
DB00181	D000437
DB00904	D000437
DB01104	D000437
DB00273	D000437
DB00619	D046152
DB01268	D046152
DB00843	D000544
DB00674	D000544
DB00989	D000544
DB01043	D000544
DB00635	D000542
DB00864	D001528
DB01080	C535803
DB00997	D012514
DB00694	D012514
DB00997	D012516
DB00515	D012516
DB01181	D012516
DB00563	D012516
DB00583	D008052
DB00741	D002819
DB00443	D002819
DB01234	D002819
DB00620	D002819
DB00959	D002819
DB00860	D002819
DB01380	D002819

Table S1 Continued

DrugBank ID	Disease MeSH ID
DB00997	D002813
DB00851	D002813
DB01181	D002813
DB00619	D002813
DB01268	D002813
DB00175	D005271
DB01095	D005271
DB01076	D005271
DB01098	D005271
DB00227	D005271
DB00641	D005271
DB00630	D005271
DB00399	D005271
DB00531	D009182
DB00997	D009182
DB00541	D009182
DB00635	D009182
DB00307	D009182
DB03904	D007645
DB00162	D007645
DB00819	C564234
DB00195	C564234
DB01194	C564234
DB00521	C564234
DB00869	C564234
DB00654	C564234
DB01210	C564234
DB01085	C564234
DB00373	C564234
DB00287	C564234
DB00443	D011695
DB00451	C536648
DB00348	D020176
DB00451	C562769
DB00158	D008591
DB00608	D008288

Table S1 Continued

DrugBank ID	Disease MeSH ID
DB01087	D008288
DB00468	D008288
DB00358	D008288
DB01586	D015209
DB01393	D015209
DB00951	D014376
DB01045	D014376
DB01045	D002779
DB01586	D002779
DB01599	D014973
DB00635	D001172
DB00563	D001172
DB00795	D001172
DB01097	D001172
DB01017	D001172
DB00035	D018500
DB00583	D056693
DB01586	D003550
DB00847	D003554
DB00682	C563039
DB00121	D028921
DB00121	D028922
DB00571	D018879
DB00343	D018879
DB00640	D018879
DB00715	D016584
DB01104	D016584
DB00285	D016584
DB00458	D016584
DB00328	D006073
DB01394	D006073
DB00437	D006073
DB00635	D006073
DB00605	D006073
DB00945	D009080
DB00136	C564005

Table S1 Continued

DrugBank ID	Disease MeSH ID
DB00515	D009959
DB00544	D009959
DB00884	D010024
DB00481	D010024
DB00915	D007251
DB00811	D007251
DB00558	D007251
DB00198	D007251
DB00515	D012468
DB01229	D012468
DB00441	D012468
DB01204	D012468
DB00361	D012468
DB00997	D012468
DB00694	D012468
DB00445	D012468
DB01177	D012468
DB00385	D012468
DB00619	D012468
DB00317	D012468
DB01259	D012468
DB00165	C536254
DB00668	D000707
DB01001	D000707
DB01075	D000707
DB00863	D000707
DB00635	D000707
DB00959	D000707
DB00451	C562771
DB00451	C562770
DB00515	D004938
DB00544	D004938
DB01229	D004938
DB00583	C538167
DB00675	D001943
DB01217	D001943

Table S1 Continued

DrugBank ID	Disease MeSH ID
DB01006	D001943
DB00990	D001943
DB00997	D001943
DB00531	D001943
DB01248	D001943
DB01229	D001943
DB00860	D005923
DB00091	D005923
DB00531	D005923
DB00181	D015419
DB01219	D015419
DB00697	D015419
DB01080	D013036
DB00531	D014890
DB00635	D014890
DB00993	D014890
DB00121	C537658
DB00313	D018887
DB00349	D018887
DB01068	D018887
DB00564	D018887
DB00635	D018887
DB00230	D009437
DB00321	D009437
DB00476	D009437
DB01047	D017449
DB01013	D017449
DB00620	D017449
DB00288	D017449
DB00547	D017449
DB01260	D017449
DB00158	D016135
DB00635	D017681
DB01005	D017681
DB00619	D017681
DB00515	D055752

Table S1 Continued

DrugBank ID	Disease MeSH ID
DB00773	D055752
DB00165	D006712
DB00541	D006391
DB00290	D006391
DB00531	D006391
DB00571	D006391
DB00997	D006394
DB00544	D006394
DB01181	D006394
DB00515	D006394
DB01229	D006394
DB00694	D006394
DB00445	D006394
DB01177	D006394
DB00385	D006394
DB00515	D007822
DB00544	D007822
DB01229	D007822
DB01248	D007822
DB00290	D010412
DB00515	D010412
DB00563	D010412
DB00544	D010412
DB01204	D009103
DB00993	D009103
DB00682	D020152
DB00175	D006938
DB01076	D006938
DB01098	D006938
DB01076	D006937
DB00973	D006937
DB00175	D006937
DB00641	D006937
DB00653	C537153
DB00190	D020734
DB01235	D020734

Table S1 Continued

DrugBank ID	Disease MeSH ID
DB00494	D020734
DB00323	D020734
DB00413	D020734
DB00268	D020734
DB00248	D020734
DB00714	D020734
DB01367	D020734
DB01037	D020734
DB00915	D020734
DB00860	D019693
DB00993	D019693
DB00544	D015179
DB01101	D015179
DB00762	D015179
DB00531	D010235
DB00541	D010235
DB00851	D010235
DB00421	D053579
DB00384	D053579
DB00700	D053579
DB01013	D003876
DB00596	D003876
DB00443	D003876
DB00547	D003876
DB01047	D003876
DB00588	D003876
DB00620	D003876
DB00741	D003876
DB00764	D003876
DB00091	D003876
DB00993	D003876
DB00563	D003876
DB00688	D003876
DB00523	D003876
DB00860	D003876
DB00741	C565974

Table S1 Continued

DrugBank ID	Disease MeSH ID
DB01234	C565974
DB01045	D002006
DB00254	D002006
DB01137	D002006
DB01208	D002006
DB00759	D002006
DB00860	D009404
DB00091	D009404
DB00531	D009404
DB00541	D054218
DB00635	D054218
DB00694	D054218
DB00563	D054218
DB00531	D054218
DB00997	D054218
DB01234	D054218
DB00987	D054218
DB01033	D054218
DB00125	C537475
DB00125	C565375
DB00181	C536833
DB00140	C536833
DB00583	C536833
DB00811	D007835
DB00155	C562687
DB00136	C562688
DB01005	D011087
DB00882	D011085
DB00331	D011085
DB00140	C535737
DB00338	D016481
DB00213	D016481
DB01129	D016481
DB00736	D016481
DB01211	D016481

Table S1 Continued

DrugBank ID	Disease MeSH ID
DB00653	C567127
DB00379	C538353
DB00819	C538353
DB00515	D002583
DB01229	D002583
DB01030	D002583
DB00441	D002583
DB00441	D001749
DB00515	D001749
DB00563	D001749
DB00570	D001749
DB00997	D001749
DB00350	D056734
DB00446	D014435
DB01059	D014435
DB01208	D014435
DB00262	D005910
DB01168	D005910
DB00541	D005910
DB00829	D003294
DB00564	D003294
DB01174	D003294
DB00252	D003294
DB00313	D003294
DB00360	D017827
DB00555	D017827
DB01273	D017827
DB00445	D013274
DB00515	D013274
DB00544	D013274
DB01248	D013274
DB00451	C564766
DB00501	D010437
DB00670	D010437
DB00338	D010437

Table S1 Continued

DrugBank ID	Disease MeSH ID
DB00564	C563475
DB00993	C536512
DB01033	C536512
DB00666	C562787
DB00104	D000172
DB00248	D000172
DB00675	D000172
DB00715	D010698
DB00176	D010698
DB01217	D004715
DB01006	D004715
DB00014	D004715
DB00666	D004715
DB00254	D008554
DB00446	D008554
DB00537	D008554
DB01165	D008554
DB00544	D005706
DB00997	D005706
DB00441	D005706
DB00502	D005879
DB01100	D005879
DB00158	D001139
DB00415	D008088
DB01015	D008088
DB00798	D008088
DB01393	D006950
DB00175	D006950
DB01599	D006950
DB00973	D006950
DB00544	D001005
DB00515	D001005
DB00997	D001005
DB00741	D003882
DB00443	D003882

Table S1 Continued

DrugBank ID	Disease MeSH ID
DB00715	D009771
DB00472	D009771
DB01242	D009771
DB00502	D009771
DB00734	D009771
DB00091	D013262
DB00741	C564577
DB01234	C564577
DB00531	D012512
DB00541	D012512
DB00997	D012512
DB00851	D012512
DB00970	D012512
DB01181	D012512
DB00773	D012512
DB00440	D006105
DB01167	D006105
DB00936	D010916
DB00982	D010916
DB00459	D010916
DB02300	D010916
DB00563	D010916
DB00091	D010916
DB00993	D010916
DB00740	D000690
DB00906	D000690
DB00136	D006962
DB01012	D006962
DB00136	C562794
DB00421	D006929
DB00700	D006929
DB01073	D015451
DB00531	D015451
DB00531	D015459
DB00541	D015459

Table S1 Continued

DrugBank ID	Disease MeSH ID
DB00997	D015459
DB00635	D015459
DB00630	D010001
DB01077	D010001
DB00997	D018227
DB00851	D018227
DB01181	D018227
DB00619	D018227
DB01268	D018227
DB00531	D002051
DB00541	D002051
DB00997	D002051
DB00635	D002051
DB00254	D002690
DB00207	D002690
DB01165	D002690
DB01045	D002690
DB01611	D008180
DB00959	D008180
DB00635	D008180
DB00531	D008180
DB00688	D008180
DB00993	D008180
DB00531	D008181
DB00635	D008181
DB00864	D008181
DB01611	D008181
DB00615	D009165
DB01085	D012859
DB00185	D012859
DB00091	D012859
DB00997	D013584
DB00851	D013584
DB01181	D013584
DB00619	D013584

Table S1 Continued

DrugBank ID	Disease MeSH ID
DB01268	D013584
DB00983	D029424
DB00938	D029424
DB00986	D029424
DB01077	D018212
DB00720	D018212
DB00282	D018212
DB00630	D018212
DB01133	D018212
DB00710	D018212
DB00884	D018212
DB00399	D018212
DB00745	D009290
DB00422	D009290
DB00182	D009290
DB00579	D009290
DB00230	D051474
DB00959	D009902
DB01259	D016518
DB00244	D003093
DB01222	D003093
DB00545	D009157
DB01122	D009157
DB01010	D009157
DB00443	D009157
DB00864	D009157
DB00091	D009157
DB00571	D006111
DB00763	D006111
DB00550	D006111

Table S2 Results of drug-disease pairs in the top group

Drug	Rank	Disease MeSH ID	Disease name	Z score	Verified information	Purpose	Article validation	Article name	Journal
**Sirolimus**	1	D008106	Liver cirrhosis, experimental	36.105	NA		Yes	Low-dose oral rapamycin treatment reduces fibrogenesis, improves liver function, and prolongs survival in rats with established liver cirrhosis	Journal of Hepatology
	2	C562729	Esophageal squamous cell carcinoma	33.5323	NA		Yes	An activated mTOR/ p70S6K signaling pathway in esophageal squamous cell carcinoma cell lines and inhibition of the pathway by rapamycin and siRNA against mTOR	Cancer Letters
	3	D001254	Astrocytoma	16.6658	KEGG, DrugBank, ClinicalTrials	Treatment			
	4	D008654	Mesothelioma	6.6156	NA		Yes	Combined treatment with cisplatin and sirolimus to enhance cell death in human mesothelioma	The Journal of Thoracic and Cardiovascular Surgery
	5	D006528	Carcinoma, hepatocellular	4.5865	DrugBank, ClinicalTrials	Treatment			
	6	D012559	Schizophrenia	4.5449	NA				
	7	D006973	Hypertension	4.5395	DrugBank, ClinicalTrials	Treatment			
	8	D002292	Carcinoma, renal cell	4.4973	DrugBank, ClinicalTrials	Treatment			
	9	D010051	Ovarian neoplasms	4.4841	ClinicalTrials	Treatment			
	10	D000230	Adenocarcinoma	4.4709	ClinicalTrials	Treatment			
	11	D054218	Precursor T-cell lymphoblastic leukemia-lymphoma	4.4539	ClinicalTrials	Treatment			
	12	D020522	Lymphoma, mantle-cell	4.45	DrugBank, ClinicalTrials	Treatment			

Table S2 Continued

Drug	Rank	Disease MeSH ID	Disease name	Z score	Verified information	Purpose	Article validation	Article name	Journal
	13	D017379	Hypertrophy, left ventricular	4.443	NA		Yes	Effect of sirolimus on left ventricular hypertrophy in kidney transplant recipients: a 1-year nonrandomized controlled trial	American Journal of Kidney Diseases
	14	D002583	Uterine cervical neoplasms	4.4427	NA				
	15	D054220	Malformations of cortical development	4.4297	NA				
	16	D020258	Neurotoxicity syndromes	4.4249	NA				
	17	D018288	Carcinoma, small cell	4.4208	NA				
	18	C537067	Focal cortical dysplasia of Taylor	4.4181	NA				
	19	D011471	Prostatic neoplasms	2.4137	ClinicalTrials	Treatment			
	20	D017202	Myocardial ischemia	2.2391	NA		Yes	Rapamycin protects against myocardial ischemia–reperfusion injury through JAK2–STAT3 signaling pathway	Journal of Molecular and Cellular Cardiology
**Metformin**	1	D013274	Stomach neoplasms	44.1332	NA		Yes	The antidiabetic drug metformin inhibits gastric cancer cell proliferation *in vitro* and *in vivo*	Molecular Cancer Therapeutics
	2	D018450	Disease progression	44.0338	ClinicalTrials	Treatment			
	3	D011085	Polycystic ovary syndrome	10.8756	KEGG, DrugBank, ClinicalTrials	Treatment			
	4	D003924	Diabetes mellitus, type 2	10.8566	KEGG, DrugBank, ClinicalTrials	Treatment			
	5	D008106	Liver cirrhosis, experimental	0.1559	NA				
	6	D001943	Breast neoplasms	0.1404	DrugBank, ClinicalTrials	Prevention			

Table S2 Continued

Drug	Rank	Disease MeSH ID	Disease name	Z score	Verified information	Purpose	Article validation	Article name	Journal
	7	D011471	Prostatic neoplasms	0.1212	DrugBank, ClinicalTrials	Treatment			
	8	D009765	Obesity	0.1122	DrugBank, ClinicalTrials	Treatment			
	9	D006528	Carcinoma, hepatocellular	0.0784	DrugBank, ClinicalTrials	Treatment			
	10	D007333	Insulin resistance	0.0585	DrugBank, ClinicalTrials	Treatment			
	11	D002289	Carcinoma, non-small-cell lung	0.0532	DrugBank, ClinicalTrials	Treatment			
	12	D015464	Leukemia, myelogenous, chronic, BCR-ABL positive	0.041	NA				
	13	D004715	Endometriosis	0.0409	NA		Yes	Metformin is a potent inhibitor of endometrial cancer cell proliferation—implications for a novel treatment strategy	Gynecologic Oncology
	14	D003528	Carcinoma, adenoid cystic	0.0346	NA				
	15	D018149	Glucose intolerance	0.0338	DrugBank, ClinicalTrials	Basic Science			
	16	D002545	Brain ischemia	0.0297	NA				
	17	D008224	Lymphoma, follicular	0.027	NA		Yes	Therapeutic metformin/AMPK activation blocked lymphoma cell growth *via* inhibition of mTOR pathway and induction of autophagy	Cell Death & Disease
	18	D010051	Ovarian neoplasms	0.0265	ClinicalTrials	Treatment			
	19	C563663	Immunodeficiency due to defect in MAPBP-interacting protein	0.0262	NA				
	20	C565485	Glycogen storage disease 0, liver	0.0258	NA				

Table S2 Continued

Drug	Rank	Disease MeSH ID	Disease name	Z score	Verified information	Purpose	Article validation	Article name	Journal
**Itraconazole**	1	D002177	Candidiasis	190.011	KEGG, DrugBank, ClinicalTrials	Treatment			
	2	D006105	Granulomatous disease, chronic	189.774	KEGG, ClinicalTrials	Treatment			
	3	D008106	Liver cirrhosis, experimental	5.87	NA				
	4	D011471	Prostatic neoplasms	3.3631	DrugBank, ClinicalTrials	Treatment			
	5	D006528	Carcinoma, hepatocellular	1.908	NA				
	6	D008175	Lung neoplasms	1.2561	ClinicalTrials	Treatment			
	7	D009765	Obesity	1.2313	NA				
	8	D001943	Breast neoplasms	1.2022	DrugBank, ClinicalTrials	Treatment			
	9	D013274	Stomach neoplasms	1.1423	NA		Yes	Itraconazole induces apoptosis and cell cycle arrest *via* inhibiting Hedgehog signaling in gastric cancer cells	Journal of Experimental & Clinical Cancer Research
	10	D008114	Liver neoplasms, experimental	1.0895	NA				
	11	D000544	Alzheimer disease	0.8671	DrugBank, ClinicalTrials	Treatment			
	12	D002779	Cholestasis	0.8584	NA				
	13	D004715	Endometriosis	0.8474	NA		Yes	Itraconazole inhibits AKT/mTOR signaling and proliferation in endometrial cancer cells	Anticancer Research
	14	D056486	Chemical and drug induced liver injury	0.7955	NA				
	15	D010381	Pelger-Huet anomaly	0.7805	NA				
	16	D008325	Mammary neoplasms, experimental	0.7786	NA				

Table S2 Continued

Drug	Rank	Disease MeSH ID	Disease name	Z score	Verified information	Purpose	Article validation	Article name	Journal
	17	D011778	Q fever	0.7616	NA				
	18	D015179	Colorectal neoplasms	0.7442	NA				
	19	D007676	Kidney failure, chronic	0.7379	NA				
	20	D011085	Polycystic ovary syndrome	0.7279	DrugBank, ClinicalTrials	Basic Science			
**Risperidone**	1	D007022	Hypotension	8.0016	DrugBank	NA			
	2	D012559	Schizophrenia	6.845	DrugBank, ClinicalTrials	Treatment			
	3	D009771	Obsessive-compulsive disorder	6.266	KEGG, DrugBank, ClinicalTrials	Treatment			
	4	D006332	Cardiomegaly	4.7158	NA				
	5	D019969	Amphetamine-related disorders	4.3576	NA				
	6	D006948	Hyperkinesis	3.7844	ClinicalTrials	Treatment			
	7	D005355	Fibrosis	3.6146	NA		Yes	Effects of clozapine, chlorpromazine and risperidone treatment on the liver fibrosis markers	Journal of Radioimmunology
	8	D012640	Seizures	3.5542	DrugBank	NA			
	9	D019970	Cocaine-related disorders	3.0679	DrugBank, ClinicalTrials	Treatment			
	10	D010146	Pain	2.9074	DrugBank	NA			
	11	D013375	Substance withdrawal syndrome	2.8833	NA				
	12	D003921	Diabetes mellitus, experimental	2.5751	NA				
	13	D008103	Liver cirrhosis	2.5446	NA				

Table S2 Continued

Drug	Rank	Disease MeSH ID	Disease name	Z score	Verified information	Purpose	Article validation	Article name	Journal
	14	D006333	Heart failure	2.542	NA				
	15	D013981	Tic disorders	2.5198	NA		Yes	Risperidone treatment of children and adolescents with chronic tic disorders: a preliminary report	Child & Adolescent Psychiatry
	16	D020257	Ventricular remodeling	2.5156	NA				
	17	D009765	Obesity	2.0929	DrugBank	Screening			
	18	D008569	Memory disorders	1.9297	NA				
	19	D008607	Intellectual disability	1.9045	ClinicalTrials	Treatment			
	20	D008171	Lung diseases	1.8888	NA				

Note: The verified information column indicates the information source (KEGG, DrugBank clinical indication, or ClinicalTrials).

Table S3 Results of drug-disease pairs in the random group

Drug	Rank	Disease MeSH ID	Disease name	Z score	Verified information	Purpose	Article validation	Article name	Journal
**Sirolimus**	2121	D006105	Granulomatous disease, chronic	−0.0563	NA		Yes	Sirolimus as an alternative treatment in patients with granulomatous-lymphocytic lung disease and humoral immunodeficiency with impaired regulatory T cells	Respiratory Medicine Case Reports
	2164	C538258	ATR-X syndrome	−0.0563	NA				
	682	C537533	Seckel syndrome 1	−0.0529	NA				
	2372	C566869	Night blindness, congenital stationary, autosomal dominant 2	−0.0565	NA				
	1148	C567656	Cerebellar ataxia, mental retardation, and disequilibrium syndrome 2	−0.0548	NA				
	2303	C567275	Craniodiaphyseal dysplasia, autosomal dominant	−0.0564	NA				
	226	D008569	Memory disorders	−0.0419	NA		Yes	Rapamycin-sensitive late-LTP is enhanced in the hippocampus of IL-6 transgenic mice	Neuroscience
	341	D013163	Splenomegaly	−0.048	NA		Yes	Rapamycin reverses splenomegaly and inhibits tumor development in a transgenic model of Epstein-Barr virus-related Burkitt's lymphoma	Molecular Cancer Therapeutics
	3101	D056660	Hereditary autoinflammatory diseases	−0.057	NA				
	3313	D041781	Jaundice, obstructive	−0.0572	NA				
	1621	D014648	Varicose veins	−0.0557	NA				
	2515	C562626	Ehlers-Danlos syndrome, type VIII	−0.0566	NA				
	777	C536623	Scalp ear nipple syndrome	−0.0534	NA				

Table S3 Continued

Drug	Rank	Disease MeSH ID	Disease name	Z score	Verified information	Purpose	Article validation	Article name	Journal
	3135	C564485	Glycogen storage disease, type IXD	−0.0571	NA		Yes	Preclinical development of new therapy for glycogen storage diseases	Current Gene Therapy
	2710	D010013	Osteogenesis imperfecta	−0.0568	NA		Yes	Rapamycin promotes osteogenesis under inflammatory conditions	Molecular Medicine Reports
	86	C538231	Adenocarcinoma of lung	−0.0113	DrugBank	NA			
	3590	D012178	Retinopathy of prematurity	−0.0574	NA		Yes	Anti-angiogenic effects of mammalian target of rapamycin inhibitors in a mouse model of oxygen-induced retinopathy	Biological & Pharmaceutical Bulletin
	3105	D002549	Diffuse cerebral sclerosis of Schilder	−0.057	NA				
	1518	C567482	Hypospadias 1, X-linked	−0.0555	NA				
	3939	D018382	Thyroid hormone resistance syndrome	−0.0578	NA				
**Metformin**	1158	C536739	Wolcott-Rallison syndrome	−0.0276	NA				
	3867	D010201	Panniculitis, nodular nonsuppurative	−0.029	NA				
	958	D008067	Lipoma	−0.0274	NA				
	3370	D009471	Neuromyelitis optica	−0.0288	NA				
	2359	D006869	Hydronephrosis	−0.0285	NA				
	350	D012220	Rhinitis	−0.0253	NA				
	3561	D009212	Myoglobinuria	−0.0289	NA				
	2720	D009056	Mouth abnormalities	−0.0286	NA				
	1155	C563614	Long QT syndrome 2	−0.0276	NA				
	110	D003866	Depressive disorder	−0.0139	ClinicalTrials				
	1151	D046351	Protoporphyria, erythropoietic	−0.0276	NA				

Table S3 Continued

Drug	Rank	Disease MeSH ID	Disease name	Z score	Verified information	Purpose	Article validation	Article name	Journal
	251	D012516	Osteosarcoma	−0.0235	NA		Yes	Inhibition of LCMR1 and ATG12 by demethylation-activated miR-570-3p is involved in the anti-metastasis effects of metformin on human osteosarcoma	Cell Death & Disease
	1273	D016553	Purpura, thrombocytopenic, idiopathic	−0.0277	NA				
	183	D010580	Peutz-Jeghers syndrome	−0.0214	NA				
	4044	C536601	Amaurosis congenita of Leber, type 2	−0.0291	NA				
	3109	C563676	Retinitis pigmentosa 33	−0.0288	NA		Yes	Rescue of mutant rhodopsin traffic by metformin-induced AMPK activation accelerates photoreceptor degeneration	Human Molecular Genetics
	3171	C565188	Spinocerebellar ataxia, autosomal recessive 8	−0.0288	NA				
	2798	C566882	Surfactant metabolism dysfunction, pulmonary, 1	−0.0287	NA				
	2746	D008336	Mandibular diseases	−0.0286	NA				
	2690	C567709	Muscular dystrophy, congenital, due to integrin alpha-7 deficiency	−0.0286	NA		Yes	Effects of single and combined metformin and L-citrulline supplementation on L-arginine-related pathways in Becker muscular dystrophy patients: possible biochemical and clinical implications	Amino Acids
**Itraconazole**	2105	C576976	Hypothyroidism, congenital, nongoitrous, 1	−0.1167	NA				
	647	D011686	Purine-pyrimidine metabolism, inborn errors	−0.1011	NA				
	4089	C536602	Amaurosis congenita of Leber, type 5	−0.1223	NA				

Table S3 Continued

Drug	Rank	Disease MeSH ID	Disease name	Z score	Verified information	Purpose	Article validation	Article name	Journal
	2657	C566169	Cardiomyopathy, familial hypertrophic, 4	−0.1178	NA				
	1252	C565822	Congenital cataracts, facial dysmorphism, and neuropathy	−0.114	NA				
	2157	C535420	Charcot-Marie-Tooth disease, type 4b1	−0.1168	NA				
	609	D016736	Antiphospholipid syndrome	−0.0993	NA				
	3037	C538157	Blau syndrome	−0.1196	NA				
	2386	C567680	Waardenburg syndrome, type 4b	−0.1173	NA				
	1619	C536025	Marshall syndrome	−0.1155	NA				
	3961	D000033	Abortion, threatened	−0.1216	NA				
	4037	D019595	Severe dengue	−0.1219	NA				
	2169	C563408	Epidermolysis bullosa simplex, autosomal recessive	−0.1168	NA				
	2726	C537194	Lethal congenital contracture syndrome 1	−0.1181	NA				
	2293	C567514	Long QT syndrome 10	−0.1171	NA				
	1569	C567195	Exocrine pancreatic insufficiency, dyserythropoietic anemia, and calvarial hyperostosis	−0.1154	NA				
	2504	C538247	Amish lethal microcephaly	−0.1175	NA				
	2989	D052919	Refsum disease, infantile	−0.1193	NA				
	3243	D000402	Airway obstruction	−0.1202	NA				
	3333	C563669	Deafness, autosomal recessive 68	−0.1204	NA				

Table S3 Continued

Drug	Rank	Disease MeSH ID	Disease name	Z score	Verified information	Purpose	Article validation	Article name	Journal
**Risperidone**	3020	C536271	Ichthyosis prematurity syndrome	−0.031	NA				
	3355	C562440	Hypophosphatasia, childhood	−0.0311	NA				
	2962	C535684	Ring dermoid of cornea	−0.031	NA				
	3723	D009402	Nephrosis, lipoid	−0.0312	NA				
	1553	D008580	Meningism	−0.0302	NA				
	3116	C564520	Retinitis pigmentosa 3	−0.031	NA				
	419	D003092	Colitis	−0.0278	NA				
	1128	C562735	Osseous heteroplasia, progressive	−0.0298	NA				
	1135	C563181	Histiocytoma, angiomatoid fibrous	−0.0298	NA				
	2111	D058489	46, XX disorders of sex development	−0.0306	NA				
	2005	C537989	Charcot-Marie-Tooth disease, type 2b	−0.0305	NA				
	1091	C535736	Encephalocraniocutaneous lipomatosis	−0.0297	NA				
	1121	C564593	Gaze palsy, familial horizontal, with progressive scoliosis	−0.0298	NA				
	1062	D007926	Lesch-Nyhan syndrome	−0.0297	NA		Yes	Evaluation of risperidone in the neonatal 6-hydroxydopamine model of Lesch-Nyhan syndrome	Pharmacology Biochemistry & Behavior
	2418	C566910	Renal tubular acidosis, distal, with hemolytic anemia	−0.0307	NA				
	1011	D011225	Pre-eclampsia	−0.0296	NA				

Table S3 Continued

Drug	Rank	Disease MeSH ID	Disease name	Z score	Verified information	Purpose	Article validation	Article name	Journal
	1822	C562710	Diabetes mellitus, insulin-resistant, with acanthosis nigricans	−0.0304	NA				
	1398	C564093	Myopathy, X-linked, with excessive autophagy	−0.0301	NA				
	3019	C564714	Spondyloepimetaphyseal dysplasia, X-linked	−0.031	NA				
	626	D014376	Tuberculosis	−0.0291	NA				

Note: The verified information column indicates the information source (KEGG, DrugBank clinical indication, or ClinicalTrials).

Table S4 The compounds showing high similarity to nifedipine or nortriptyline, on the basis of their gene expression profiles

Drug	Compound	Activity	Article name	Journal
Nifedipine	Rilmenidine	Suppression of proliferation and promotion of apoptosis *via* the mitochondrial pathway in human leukemic K562 cells	Rilmenidine suppresses proliferation and promotes apoptosis *via* the mitochondrial pathway in human leukemic K562 cells	European Journal of Pharmaceutical Sciences
	Nomilin	Anti-tumor and immunomodulatory effects *in vivo*	Nomilin inhibits metastasis *via* induction of apoptosis and regulates the activation of transcription factors and the cytokine profile in B16F-10 cells	Integrative Cancer Therapies
			Nomilin inhibits tumor-specific angiogenesis by downregulating VEGF, NO and proinflammatory cytokine profile and also by inhibiting the activation of MMP-2 and MMP-9	European Journal of Pharmacology
			Limonoids and their anti-proliferative and anti-aromatase properties in human breast cancer cells	Food & Function
			Inhibition of tumor progression by naturally occurring terpenoids	Pharmaceutical Biology
	AICA-ribonucleotide	Induction of apoptosis and programmed necrosis in prostate cancer cells	AICAR induces AMPK-independent programmed necrosis in prostate cancer cells	Biochemical and Biophysical Research Communications
			Activation of AMP-kinase by AICAR induces apoptosis of DU-145 prostate cancer cells through generation of reactive oxygen species and activation of c-Jun N-terminal kinase	International Journal of Oncology
			AMP-activated protein kinase activators can inhibit the growth of prostate cancer cells by multiple mechanisms	Biochemical and Biophysical Research Communications
	CGP-57380	Suppression of eIF4E phosphorylation by Mnk inhibitors sensitization of CRPC cells to mTOR and AR inhibitors	The Androgen Receptor is a negative regulator of eIF4E Phosphorylation at S209: Implications for the use of mTOR inhibitors in advanced prostate cancer	Oncogene
	TPCA-1	Direct dual inhibition of STAT3 and NF-κB and regression of mutant EGFR-associated human non-small cell lung cancers	TPCA-1 is a direct dual inhibitor of STAT3 and NF-κB and regresses mutant EGFR-associated human non-small cell lung cancers	Molecular Cancer Therapeutics
	Benzopyrene	Induction of cell death, DNA strand breaks, and cell cycle arrest in the DU145 human prostate carcinoma cell line	Induction of cell death, DNA strand breaks, and cell cycle arrest in DU145 human prostate carcinoma cell line by benzo[a]pyrene	International Journal of Environmental Research and Public Health

Table S4 Continued

Drug	Compound	Activity	Article name	Journal
	Indirubin	Inhibition of prostate tumor growth through inhibiting tumor angiogenesis	Indirubin inhibits tumor growth by antitumor angiogenesis *via* blocking VEGFR2-mediated JAK/STAT3 signaling in endothelial cell	International Journal of Cancer
Nortriptyline	Sertraline	Induction of [Ca(2+)](i) rise in human PC3 prostate cancer cells	The mechanism of sertraline-induced [Ca(2+)](i) rise in human PC3 prostate cancer cells	Basic & Clinical Pharmacology & Toxicology
	Tetrindole			
	RS-17053			
	Triflupromazine			
	Terfenadine	Induction of anti-proliferative and apoptotic activities in human hormone-refractory prostate cancer through histamine receptor-independent Mcl-1 cleavage and Bak up-regulation	Terfenadine induces anti-proliferative and apoptotic activities in human hormone-refractory prostate cancer through histamine receptor-independent Mcl-1 cleavage and Bak up-regulation	Naunyn-Schmiedeberg's Archives of Pharmacology
	T-98475			
	Ispinesib	Inhibition of the mitotic kinesin spindle protein, thus leading to cell cycle arrest and cell death with activity in a wide spectrum of tumor cell lines and xenografts and preliminary clinical activity in early phase I and II	Increased therapeutic potential of an experimental anti-mitotic inhibitor SB715992 by genistein in PC-3 human prostate cancer cell line	BMC Cancer
			Promising novel cytotoxic agents and combinations in metastatic prostate cancer	The Cancer Journal
	Mibefradil	Induction of cell apoptosis in leukemia and glioblastoma cell lines	T-type calcium channel antagonists, mibefradil and NNC-55-0396 inhibit cell proliferation and induce cell apoptosis in leukemia cell lines	Journal of Experimental & Clinical Cancer Research
			Inhibition of T-type calcium channels disrupts Akt signaling and promotes apoptosis in glioblastoma cells	Biochemical Pharmacology
	KB-R7943	Promotion of cell death in PCa by activating the JNK signaling pathway and blocking autophagic flux	The reverse-mode NCX1 activity inhibitor KB-R7943 promotes prostate cancer cell death by activating the JNK pathway and blocking autophagic flux	Oncotarget

References

1. Chen X, Liu MX, Yan GY. Drug-target interaction prediction by random walk on the heterogeneous network. Mol Biosyst. 2012; 8: 1970-8.
2. Luo HM, Wang JX, Li M, Luo JW, Ni P, Zhao KJ, et al. Computational drug repositioning with random walk on a heterogeneous network. IEEE-ACM Trans Comput Biol Bioinform. 2018; 16: 1890-900.
3. Edgar RS, Stangherlin A, Nagy AD, Nicoll MP, Efstathiou S, O'Neill JS, et al. Cell autonomous regulation of herpes and influenza virus infection by the circadian clock. Proc Natl Acad Sci U S A. 2016; 113: 10085-90.
4. Shannon P, Markiel A, Ozier O, Baliga NS, Wang JT, Ramage D, et al. Cytoscape: a software environment for integrated models of biomolecular interaction networks. Genome Res. 2003; 13: 2498-504.
5. Jensen LJ, Kuhn M, Stark M, Chaffron S, Creevey C, Muller J, et al. String 8—a global view on proteins and their functional interactions in 630 organisms. Nucleic Acids Res. 2009; 37: D412-6.
6. Lamb J, Crawford ED, Peck D, Modell JW, Blat IC, Wrobel MJ, et al. The connectivity map: using gene-expression signatures to connect small molecules, genes, and disease. Science. 2006; 313: 1929-35.
